# Reporting of research priorities in migraine clinical practice guidelines still need improvement—A methodological analysis

**DOI:** 10.1371/journal.pone.0361302

**Published:** 2026-10-30

**Authors:** Xuejian Yin, Yingdi Liao, Zhuo Ye, Yiyun Zhao, Shiyu An, Riai Tang, Qinyan Li, Shuting Yang, Yuze Sun, Yicheng Gao, Kai Yuan

**Affiliations:** 1 The Second Clinical Medical College, Yunnan University of Chinese Medicine, Kunming, China; 2 The First Clinical Medical College, Yunnan University of Chinese Medicine, Kunming, China; 3 College of Basic Medical Sciences, Yunnan University of Chinese Medicine, Kunming, China; Karolinska Institutet, SWEDEN

## Abstract

**Objectives:**

To systematically analyze the content and format of research priorities in clinical practice guidelines (CPGs) for migraine, providing a methodological guidance for future guideline developers and researchers.

**Methods:**

This methodological systematic analysis involved searching six databases (Embase, PubMed CNKI, VIP, CBM and Wanfang) and the official websites of 39 authoritative organizations, including headache societies, neurology associations, and guideline development bodies. Extract information such as the name, type, and quantity of research priorities. Two reviewers independently performed data retrieval, screening, and extraction. Descriptive statistics were used to calculate the absolute frequencies and proportions of research-focused items. We have registered at INPLASY with the registration number INPLASY2025120098.

**Results:**

Out of 96 included CPGs, 248 research priorities were identified. Only half (48, 50%) of the CPGs explicitly reported research priorities, and only 13.5% (13) included a dedicated section. The primary rationale for proposing these priorities was a lack of sufficient evidence (108, 43.5%). The nomenclature for research priorities varies widely, and structured reporting was found to be inadequate. Furthermore, 56% of priorities (139) focused on the effectiveness of interventions, primarily concerning calcitonin gene-related peptide (CGRP) monoclonal antibodies (20, 8.1%), angiotensin-converting enzyme inhibitors (ACEIs) or angiotensin II receptor blockers (ARBs) (18, 7.3%), and antiepileptic drugs (17, 6.9%).

**Conclusions:**

The reporting of research priorities in migraine CPGs—both in content and format—requires significant improvement. Future efforts should prioritize the systematic identification and standardized reporting of research gaps in migraine and other disease areas.

## Introduction

Migraine is a prevalent neurological disorder affecting 14% of the global population, characterized by paroxysmal headaches. It is the second leading cause of global disability, imposing a heavy burden on the global healthcare system [1,2]. While numerous clinical practice guidelines (CPGs) have been developed worldwide [3–7], many recommendations within these frameworks lack robust evidence-based support. This evidentiary gap results in significant ambiguity, presenting a formidable challenge for clinical researchers and methodological experts alike.

In the development of CPGs, some recommendations lack sufficient evidence or are based on low-quality evidence, undermining their credibility [8–10]. Therefore, many CPGs highlight areas with weak evidence and establish research priorities for specific sections. The Evidence to Decision (EtD) framework, issued by the Grading of Recommendations Assessment, Development and Evaluation (GRADE) Working Group, explicitly states that research priorities should be integrated into the formulation of CPG recommendations and consistently reported [11,12]. This approach not only directs researchers to prioritize critical research areas but also helps them avoid redundant studies in well-established fields, thus preventing the waste of resources [13]. Clinical practice guidelines are principally developed to assist clinicians and healthcare decision-makers in selecting appropriate therapeutic strategies based on available evidence. While identifying unanswered clinical questions is not their core objective, guideline expert panels are uniquely positioned to recognize gaps in existing evidence [14–16]. Therefore, systematically extracting research priorities from migraine guidelines constitutes a valuable secondary application of guideline documents.

Strictly speaking, research priorities should be evidence-based, but they should not rely solely on evidence. Similar to the formulation of recommendations, they must consider multiple factors [17–21]—often more comprehensive than those for formulating clinical recommendations. For instance, factors related to research resources must also be considered. Additional factors related to research resources and research equity must also be considered. For instance, an intervention may require substantial resources to demonstrate its efficacy but yield benefits only for a very small population, resulting in more disadvantages than advantages at the research, thereby diminishing its research priority. Moreover, guideline development manuals from various authoritative institutions have outlined the types, content, and format of research priorities [22–24].

Although some studies have explored research priorities, their methodologies have been relatively limited, and the analysis of the reporting format and content has been insufficient [21,25]. This study systematically analyzed the content of research priorities in migraine CPGs, assessed the quality of their reports, and provided valuable guidance for future migraine research and guideline development.

## Methods

### Eligibility criteria

This study included all CPGs that provided at least one recommendation for migraine management. Exclusion criteria comprised expert consensus documents, guideline development manuals, duplicate publications, and superseded versions of previously published guidelines.

### CPGs sources and searches

XY and YL collaboratively developed the search strategy and independently conducted the search. A total of six databases were searched: Embase, Pubmed, China National Knowledge Infrastructure base (CNKI), Wanfang, Chinese Biomedical Literature Database, and VIP Database for Chinese Technical Periodicals. The keywords used included “Migraine,” “Headache,” “Migraine with Aura,” “evidence-based guideline,” “clinical guidance,” and “clinical practice guidance,” among others. Additionally, we searched the official websites of 39 societies, including the Headache or Migraine Society, the Neurology Society, guideline development organizations, and authoritative comprehensive societies. The search results have been updated to March 20, 2026, before submitting the paper. A detailed description of the search strategies can be found in S1 Table.

### Definitions

Migraine CPGs were defined as guidelines containing recommendations specifically developed for migraine. Therefore, the CPGs included in this study comprise two categories: (1) Specific migraine CPGs, which focus exclusively on migraine-related recommendations; and (2) Non-specific migraine CPGs, which incorporate migraine-related recommendations as part of a broader clinical scope.

### Identification of research priorities

Terms such as “further research,” “future research,” “limitations,” and “recommendations for future research” were used to identify and define research priorities. These terms were commonly found in sections such as the evidence summaries under guideline recommendations, the Discussion, and the Future Research section. It should be emphasized that the above may involve slight differences in meaning, yet they share a consistent fundamental direction; therefore, we have all categorized them as research priorities.

### CPGs selection, data extraction and analysis

The CPGs were screened, and data extracted using a two-author back-to-back method, with any discrepancies resolved by a third-author with methodological background (YG). The items of interest in this study were extracted according to a pre-designed data extraction form: (1) basic information related to the CPGs, including publication year, issuing institution, and scope; (2) information related to research priorities, including the number, title, type, rationale, and interventions involved in the research priorities reports. Notably, we analyzed the structure of the research priorities to determine whether they included any components of the PICO (population, intervention, comparison, and outcome) framework [25]. For data analysis, descriptive statistics were used, calculating the absolute frequency and proportion of the reported items.

### Patient and public involvement

No patient involved. Ethics approval and consent to participate are not applicable to this work because the study does not include human subjects or human biological specimens.

## Results

Search resultsA total of 29,985 records were retrieved from the database, supplemented by 32 records identified through website searches. After removing duplicates, 20,184 records remained for screening. Following screening of titles and abstracts, 20,070 records were excluded, leaving 114 records for full-text eligibility assessment. Following a detailed review, 18 records were excluded, resulting in a final inclusion of 96 CPGs (Fig 1 and S2 Table).

**Fig 1:**
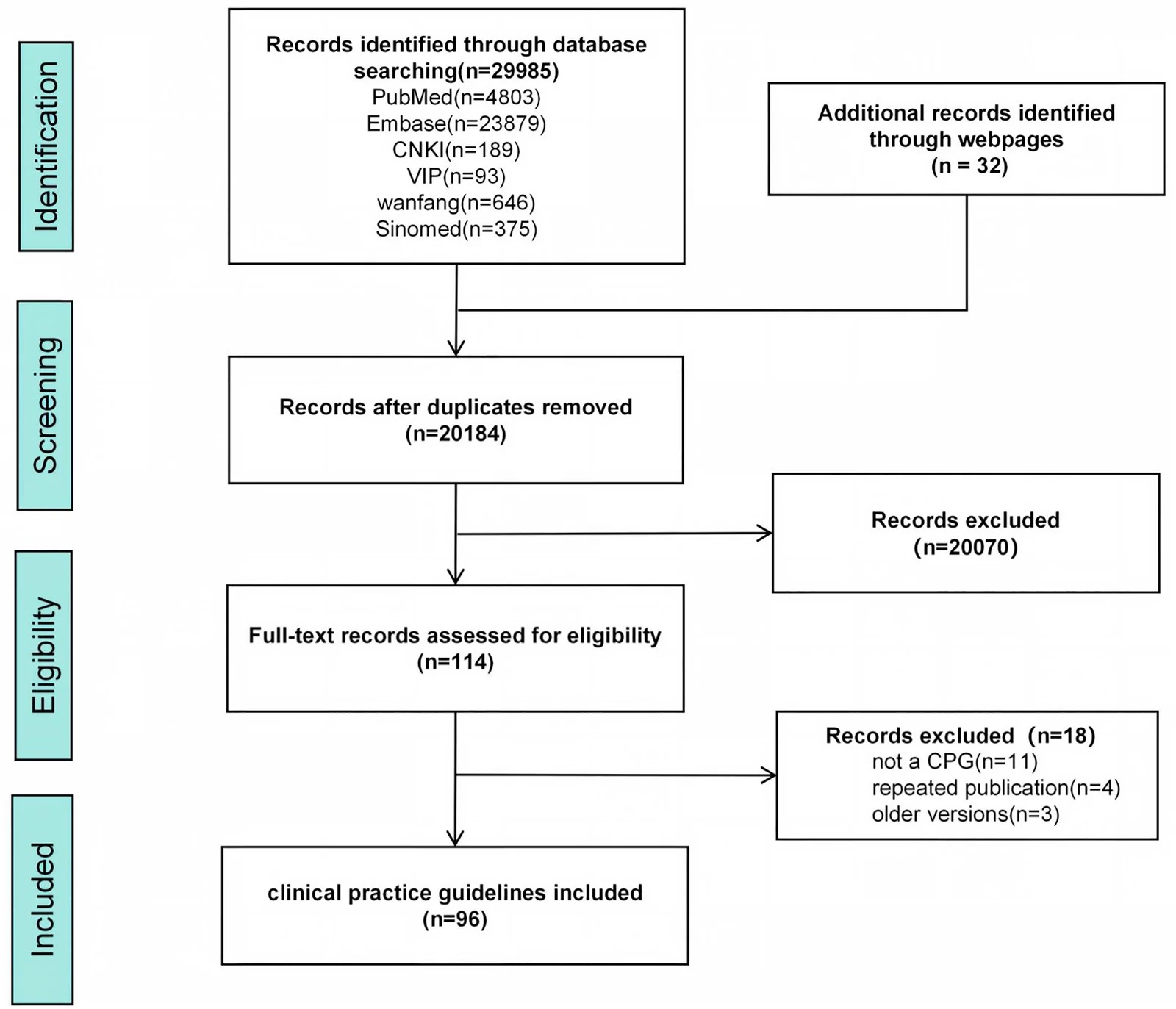
Flowchart of clinical practice guidelines screening.

### Guidelines characteristics

Of the 96 included CPGs, 63 (65.6%) were classified as specific migraine guidelines, while 33 (34.4%) were categorized as non-specific. Regarding their primary focus, 70 (72.9%) guidelines centered on disease management, whereas only 26 (27.1%) focused specifically on therapeutic interventions (Table 1).

**Table 1:** Characteristics of included clinical practice guidelines (N = 96).

Variable	Frequency	Percentage
**Scope of CPGs**		
Treatment	38	39.6
Diagnosis and treatment (or management)	21	21.9
Treatment and prevent (or management)	16	16.7
Prevent	11	11.5
Management	10	10.4
**Type of CPGs**		
specific migraine CPGs	63	65.6
non-specific migraine	33	34.4
**Focus of CPGs**		
Focus on disease	70	72.9
Focus on interventions	26	27.1
**Published year**		
2021-2025	43	44.8
2016-2020	24	25.0
2011-2015	14	14.6
2006-2010	12	12.5
Before 2000	3	3.1
**Publication organization**		
Headache or migraine society	35	36.5
Neurology society	18	18.8
Comprehensive Society or Organization	16	16.7
CPGs development organization	16	16.7
CPGs development Group	6	6.3
Others^a^	5	5.2

^a^such as government agencies or societies unrelated to headaches (e.g., the American College of Obstetricians)

CPG, clinical practice guideline.

### Basic characteristics of research priorities

Only half of the included guidelines (48, 50%) reported research priorities. Among these, 21 (21.9%) incorporated research priorities within the evidence summaries accompanying the recommendations, while only 13 (13.5%) featured a dedicated section for research priorities. Regarding the volume of identified gaps, approximately one-third (34, 35.4%) of the CPGs listed five or fewer research priorities, whereas only 3 (3.1%) identified more than ten (Table 2).

**Table 2:** Basic characteristics of research priorities (N = 96).

Categories	Clinical practice guidelines	
	Frequency	Percentage(%)
**Report research priorities**		
Reported	48	50.0
Not reported	48	50.0
**What the migraine research priorities were for**		
Specific recommendation in CPGs	19	19.8
Guidelines as a whole	24	25.0
For the entire guideline and specific recommendations	5	5.2
Not reported	48	50.0
**Location of the research priorities**		
Summary of the evidence under each recommendation	21	21.9
Independent section	13	13.5
Discussions of the CPGs	9	9.4
Independent research recommendations	4	4.2
Others^a^	1	1.0
Not reported	48	50.0
**Number of research priorities**		
≤5	34	35.4
6-10	10	10.4
11-15	1	1.0
15-20	0	0.0
≥21	2	2.1
Not reported	48	50.0

^a^Abstract.

CPG, clinical practice guideline.

### Characteristics of the report on research priorities

A total of 248 migraine-related research priorities were identified, the vast majority of which (239, 96.4%) were not presented as clinical questions. The primary reasons for proposing these research priorities (108, 43.5%) were limited or absent evidence. Regarding the nomenclature of research priorities, the most common format was “Further research/trial/study/finding/attention/confirmation/evidence/data/RCT” (71, 28.6%), followed by “Future Research/study/direction/trial” (42, 16.9%) and “Recommendations for research/Research Recommendations” (42, 16.9%). Desirable anticipated effects were the most frequently considered factors in research priorities (130, 52.4%), followed by undesirable anticipated effects (82, 33.1%) and “Only the effects was mentioned” (64, 25.8%). Other factors related to the formation of recommendations, such as health equity (6, 2.4%), values and preferences (6, 2.4%), and feasibility (4, 1.6%), were addressed less frequently. Additionally, approximately half of the research priorities contained PI structural elements, while the more comprehensive and standardized PIC or PICO structures were inadequately reported, with only 55 (22.2%) and 4 (1.6%) research priorities respectively (Table 3).

**Table 3:** Characteristics of the content and format of research priorities (N = 248).

Categories	Research priorities	
	Frequency	Percentage(%)
**Presentation forms**		
clinical question	9	3.6
Narrative sentences	239	96.4
**Are there any reasons for research priorities?**		
Yes	166	66.9
No	82	33.1
**Reasons for reporting research priorities**		
Insufficient or absent evidence	108	43.5
Low quality of evidence	35	14.1
Study design flaws	10	4.0
Clinical or research needs	20	8.1
Not reported	82	33.1
**Name of the research priorities**		
Further research/trial/study/finding/attention/confirmation/evidence/data/RCT	71	28.6
Future Research/study/direction/trial	42	16.9
Recommendations for research/research recommendations	42	16.9
Areas of insufficient/no evidence	31	12.5
Evidence gaps/research needs/limitations	26	10.5
Research/Study/trial/RCT/treatment option/clinical practice is needed/required/awaited	24	9.7
Additional/new research/trial/study	13	5.2
Need trial/study/RCT/research	9	3.6
Look forward/carry out/conduct/continue research	8	3.2
Be considered/analysed/proven/confirmed	7	2.8
Others^a^	2	0.8
**Research priorities relevant to influencing factors of recommendations**		
Desirable anticipated effects	130	52.4
Only the effects was mentioned	64	25.8
Undesirable anticipated effects	82	33.1
Resource requirements (cost)	8	3.2
Values and preferences	6	2.4
Health equity	6	2.4
Acceptability	5	2.0
Feasibility	4	1.6
Cost-effectiveness	2	0.8
**The structure of the research priorities**		
PI	114	46.0
PIC	55	22.2
P	33	13.3
PIO	14	5.6
I	12	4.8
Unstructured	11	4.4
PICO	4	1.6
PO	3	1.2
O	2	0.8

^a^The challenge ahead, Studies should try to.

PICO (population, intervention, comparison, and outcome).

### Directions and interventions in research priorities

More than half (148, 59.7%) of the migraine research priorities focused on medications, primarily including calcitonin gene-related peptide (CGRP) monoclonal antibodies, angiotensin-converting enzyme inhibitors (ACEIs) or angiotensin II receptor blockers (ARBs), and antiepileptic drugs, with the proportions being 8.1% (20), 7.3% (18), and 6.9% (17), respectively. The research directions mainly focus on efficacy (139, 56.0%), treatment plan (23, 9.3%), and research methodology issues (19, 7.7%) (Fig 2 and S3 Table).

**Fig 2:**
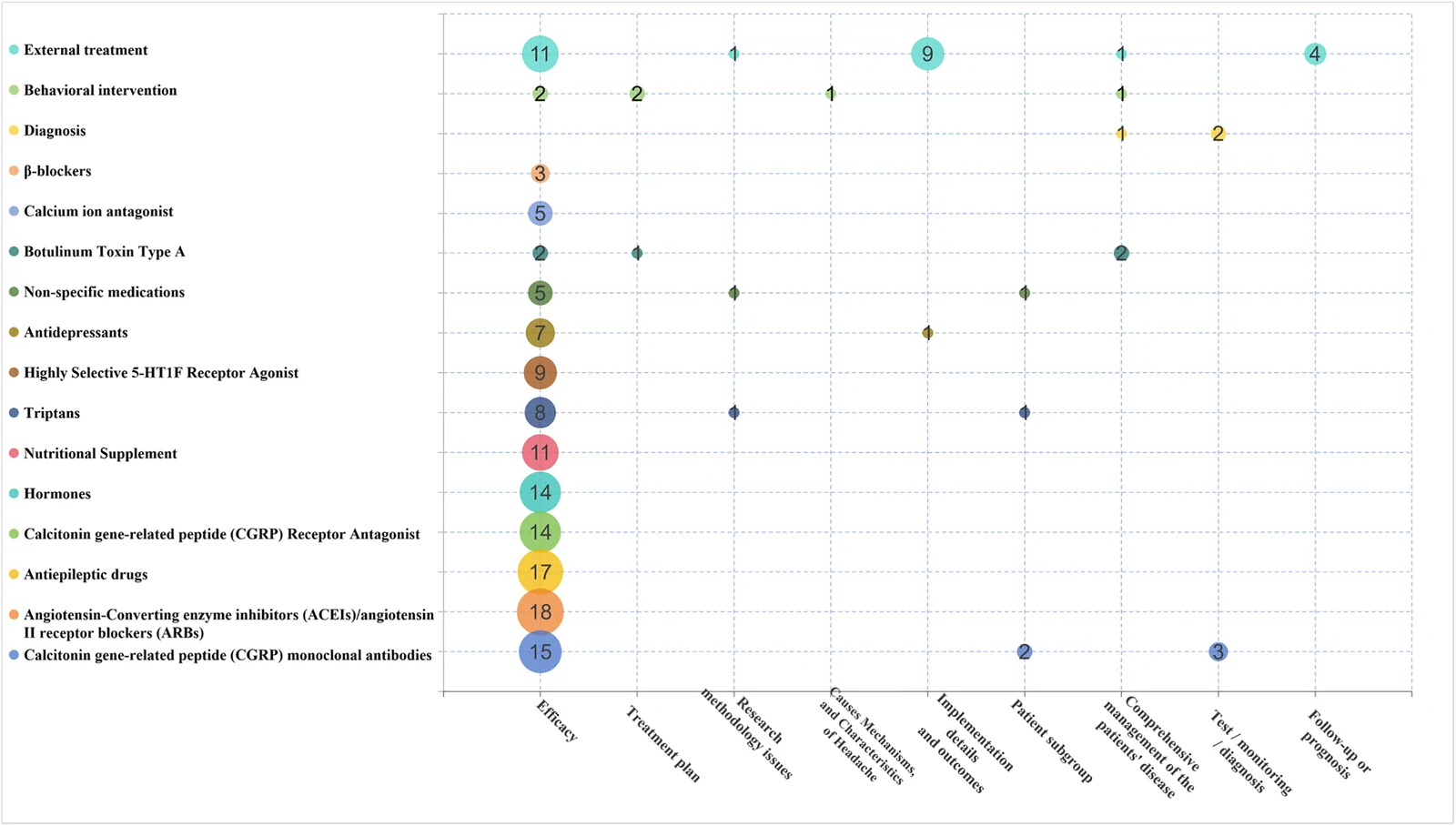
Directions and interventions in research priorities(N = 248). The X-axis represents research directions, while the Y-axis lists various migraine intervention strategies.

## Discussion

### Principal findings

This study included 96 CPGs, identifying a total of 248 research priorities. Only half of the CPGs reported research priorities, and only 13 CPGs included a distinct section on research priorities. The primary reason for proposing these priorities (108, 43.5%) was insufficient or absent evidence. The naming conventions varied, and the structured reporting was inadequate. Additionally, the research priorities primarily (139, 56%) focused on the effectiveness of interventions, which mainly involved CGRP monoclonal antibodies (20, 8.1%), ACEIs or ARBs (18, 7.3%), and antiepileptic drugs (17, 6.9%).

### Strength and limitations

As far as we know, this study is the first to systematically analyze research priorities in migraine CPGs. Through systematic retrieval, screening, and analysis, we ensured the comprehensive identification of research priorities in these guidelines. We not only summarized the reported content of migraine research priorities, such as titles, locations, and research directions, but also analyzed their format and the use of the PICO structure, providing guidance evidence for future research priority reporting.

However, our study has limitations. We believe that the evidence base for the identified research priorities varies. While weaker evidence typically demands more research, resources are limited and must be prioritized for the most urgent and significant directions. Therefore, we emphasize that weak evidence is neither a prerequisite nor a necessary condition for generating research priorities. Consequently, we did not investigate the specific reasons behind each priority. It is also important to note that different guideline development groups may define the lack of evidence or low-quality evidence differently. We respect the consensus decisions made by these groups and did not further assess their evidence-based judgments.

### Relation to previous work

Some studies suggested that research priorities should be structured according to the principles of EPICOT (evidence, population, intervention, comparison, outcome and time), with consideration given to both the type of research and the disease burden [26–28]. A previous study identified gaps in research priorities and evidence, noting that 70% of the research gaps in CPGs issued by Cystic Fibrosis Foundation were not explicitly listed as research priorities within these guidelines but rather stemmed purely from insufficient evidence supporting the formal recommendations [29]. Another study examined the content and format of research priorities reported in guidelines for knee osteoarthritis CPGs [30].

While these prior studies align with our research focus on research priorities, they fundamentally differ from this study either by failing to treat the research priorities as independent and components of guideline reports or by addressing diseases distinct from those examined here. Through analysis across various diseases, this study further clarifies the content and format of research priorities, providing a more accurate representation of their true essence. By simultaneously examining both the structured reporting standards for research priorities and their substantive thematic characteristics, our analysis advances the field and offers a more detailed and comprehensive understanding of their core attributes.

### Implications for research and guidelines

During the development of CPGs, the formulation and reporting of research priorities are crucial components that require significant effort. This process not only identifies gaps in existing evidence but also provides key research directions for CPGs users and investigators.

First, CPGs should standardize the positioning and nomenclature of research priorities to facilitate quick identification. The structure of these priorities should be standardized using frameworks such as PICO or EPICOT, which can aid in their development and enable their translation into clinical questions. This approach ensures that the implications of structural elements are maintained between clinical practice and future research, avoiding the need to reconstruct clinical questions. Second, a substantial portion of research recommendations focuses on the efficacy of interventions. Future studies should strengthen the evidence on the efficacy of various migraine treatments, particularly for drugs with high research demand. For interventions with minimal research demand, further exploration into the underlying reasons is needed to determine whether the limited number of research priorities reflects sufficient evidence, low clinical demand, or other factors. It is important to note that the absence or limited number of research priorities in certain areas does not necessarily indicate that the evidence in those fields is robust or of high quality. Third, greater attention should be given to dimensions such as health equity, feasibility, and values and preferences, with an emphasis on expanding research beyond efficacy. Additionally, methodological research priorities must receive adequate attention and reporting. Finally, areas with substantial existing evidence should be explicitly identified as research priorities to avoid redundant studies.

Regarding the methodology for guideline development, developers should recognize that guideline-specific methodologies are continuously evolving; therefore, the latest development approaches (such as GRADE approach) must be adopted to prevent diminished credibility and clinical relevance of guidelines due to methodological shortcomings. It should be emphasized that determining research priorities should not divert the core responsibility of guideline developement, which is to provide guidance for clinicians and decision-makers; whereas identifying research priorities remains a secondary objective within the guideline development. By adopting a unified reporting framework, insights from expert panels on unresolved migraine-related questions can be systematically documented, ensuring the optimization of future research directions and priorities.

## Conclusion

The content (such as dimensions considered, research directions) and format (such as degree of structuring, name, and location) of research priorities in migraine CPGs still have significant room for improvement. Future efforts should focus on the identification and reporting of research priorities in CPGs for migraine or other diseases.

## Supporting information

S1 Table Search Strategy. (DOCX)

S2 Table Included clinical practice guidelines. (DOCX)

S3 Table Directions and interventions in research priorities. (DOCX)
