# Supplementary material for: Reporting of research priorities in migraine clinical practice guidelines still need improvement—A methodological analysis
Source: PLoS One. 2026 Oct 30;21(10):e0361302. doi: 10.1371/journal.pone.0361302 (PMC13722188; doi:10.1371/journal.pone.0361302)
Supplement: Supplementary file 1 [file pone.0361302.s004.pdf]

PLOS One

Reporting of research priorities in migraine clinical practice guidelines still need improvement — A methodological analysis

--Manuscript Draft--

**Manuscript Number:**	PONE-D-26-15753
**Article Type:**	Research Article
**Full Title:**	Reporting of research priorities in migraine clinical practice guidelines still need improvement — A methodological analysis
**Short Title:**	Reporting assessment of migraine clinical practice guidelines
**Corresponding Author:**	Yicheng Gao<br>Yunnan University of Traditional Chinese Medicine<br>Kunming, Yunnan CHINA
**Keywords:**	Research priorities; Migraine; clinical decision-making; Clinical practice guidelines; Recommendations
**Abstract:**	Objectives: To systematically analyze the content and format of research priorities in clinical practice guidelines (CPGs) for migraine, providing a methodological references for future guideline developers and researchers.<br>Methods: This methodological systematic analysis involved searching six databases (Embase, PubMed CNKI, VIP, CBM and Wanfang) and the official websites of 39 authoritative organizations, including headache societies, neurology associations, and guideline development bodies. Two reviewers independently performed data retrieval, screening, and extraction. Descriptive statistics were used to calculate the absolute frequencies and proportions of research-focused items. Registered at INPLASY (INPLASY202430055).<br>Results: Out of 96 included CPGs, 248 research priorities were identified. Only half (n = 48, 50%) of the CPGs explicitly reported research priorities, and only 13.5%(n = 13) included a dedicated section. The primary rationale for proposing these priorities was a lack of sufficient evidence (n = 108, 43.5%). The nomenclature for research priorities was inconsistent, and structured reporting was found to be inadequate.<br>Furthermore, 56% of priorities (n = 139) of priorities focused on the effectiveness of interventions, primarily concerning calcitonin gene-related peptide (CGRP) monoclonal antibodies (n = 20, 8.1%), angiotensin-converting enzyme inhibitors (ACEIs) or angiotensin II receptor blockers (ARBs) (n = 18, 7.3%), and antiepileptic drugs (n = 17, 6.9%).<br>Conclusion s: The reporting of research priorities in migraine CPGs—both in content and format—requires significant improvement. Future efforts should prioritize the systematic identification and standardized reporting of research gaps in migraine and other disease areas.
**Order of Authors:**	Yicheng Gao
	Xuejian Yin
	Yingdi Liao
	Zhuo Ye
	Yiyun Zhao
	Shiyu An
	Riai Tang
	Qinyan Li
	Shuting Yang
	Yuze Sun
	Kai Yuan
**Additional Information:**	

Powered by Editorial Manager® and ProduXion Manager® from Aries Systems Corporation

Question	Response
**Financial Disclosure**<br><br>Enter a financial disclosure statement that describes the sources of funding for the work included in this submission. Review the submission guidelines for detailed requirements. View published research articles from *PLOS ONE* for specific examples.<br><br>This statement is required for submission and **will appear in the published article** if the submission is accepted. Please make sure it is accurate.<br><br>**Funded studies**<br>Enter a statement with the following details:<br>• Initials of the authors who received each award<br>• Grant numbers awarded to each author<br>• The full name of each funder<br>• URL of each funder website<br>• Did the sponsors or funders play any role in the study design, data collection and analysis, decision to publish, or preparation of the manuscript?<br><br>Did you receive funding for this work?	Yes
Please add funding details.<br>as follow-up to "**Financial Disclosure**<br><br>Enter a financial disclosure statement that describes the sources of funding for the work included in this submission. Review the submission guidelines for detailed requirements. View published research articles from *PLOS ONE* for specific examples.<br><br>This statement is required for submission and **will appear in the published article** if the submission is accepted. Please make sure it is accurate.	This work was supported by Yunnan Province's Xingdian Talents Support Program Young Scholars [grant numbers: XDYC-QNRC-2022-0269]. Funding sources have no involvement in any of the activities conducted in this article.

**Funded studies**<br>Enter a statement with the following details:<br>• Initials of the authors who received each award<br>• Grant numbers awarded to each author<br>• The full name of each funder<br>• URL of each funder website<br>• Did the sponsors or funders play any role in the study design, data collection and analysis, decision to publish, or preparation of the manuscript?<br><br>Did you receive funding for this work?"	
Please select the country of your main research funder (please select carefully as in some cases this is used in fee calculation).<br>as follow-up to "**Financial Disclosure**<br><br>Enter a financial disclosure statement that describes the sources of funding for the work included in this submission. Review the submission guidelines for detailed requirements. View published research articles from *PLOS ONE* for specific examples.<br><br>This statement is required for submission and **will appear in the published article** if the submission is accepted. Please make sure it is accurate.<br><br>**Funded studies**<br>Enter a statement with the following details:<br>• Initials of the authors who received each award<br>• Grant numbers awarded to each author<br>• The full name of each funder<br>• URL of each funder website<br>• Did the sponsors or funders play any role in the study design, data collection and analysis, decision to publish, or preparation of the manuscript?<br><br>Did you receive funding for this work?"	CHINA - CN
**Competing Interests**	All the authors state they have no conflicts of interest with regard to the content of this

Use the instructions below to enter a competing interest statement for this submission. On behalf of all authors, disclose any competing interests that could be perceived to bias this work—acknowledging all financial support and any other relevant financial or non-financial competing interests.<br><br>This statement is required for submission and **will appear in the published article** if the submission is accepted. Please make sure it is accurate and that any funding sources listed in your Funding Information later in the submission form are also declared in your Financial Disclosure statement.<br><br>View published research articles from *PLOS ONE* for specific examples.<br><br>**NO authors have competing interests**<br><br>Enter: *The authors have declared that no competing interests exist.*<br><br>**Authors with competing interests**<br><br>Enter competing interest details beginning with this statement:<br><br>*I have read the journal's policy and the authors of this manuscript have the following competing interests: [insert competing interests here]*<br><br>* typeset	article.
**Ethics Statement**<br><br>Enter an ethics statement for this submission. This statement is required if the study involved:<br><br>• Human participants<br>• Human specimens or tissue<br>• Vertebrate animals or cephalopods<br>• Vertebrate embryos or tissues	No Patient and public involvement

Powered by Editorial Manager® and ProduXion Manager® from Aries Systems Corporation

• Field research<br><br>Write "N/A" if the submission does not require an ethics statement.<br><br>General guidance is provided below. Consult the submission guidelines for detailed instructions. **Make sure that all information entered here is included in the Methods section of the manuscript.**	

Powered by Editorial Manager® and ProduXion Manager® from Aries Systems Corporation

**Format for specific study types**<br><br>**Human Subject Research (involving human participants and/or tissue)**<br>• Give the name of the institutional review board or ethics committee that approved the study<br>• Include the approval number and/or a statement indicating approval of this research<br>• Indicate the form of consent obtained (written/oral) or the reason that consent was not obtained (e.g. the data were analyzed anonymously)<br><br>**Animal Research (involving vertebrate animals, embryos or tissues)**<br>• Provide the name of the Institutional Animal Care and Use Committee (IACUC) or other relevant ethics board that reviewed the study protocol, and indicate whether they approved this research or granted a formal waiver of ethical approval<br>• Include an approval number if one was obtained<br>• If the study involved *non-human primates*, add *additional details* about animal welfare and steps taken to ameliorate suffering<br>• If anesthesia, euthanasia, or any kind of animal sacrifice is part of the study, include briefly which substances and/or methods were applied<br><br>**Field Research**<br><br>Include the following details if this study involves the collection of plant, animal, or other materials from a natural setting:<br>• Field permit number<br>• Name of the institution or relevant body that granted permission	
**Data Availability**<br><br>Authors are required to make all data underlying the findings described fully available, without restriction, and from the time of publication. PLOS allows rare exceptions to address legal and ethical concerns. See the PLOS Data Policy and FAQ for detailed information.	No - some restrictions will apply

A Data Availability Statement describing where the data can be found is required at submission. Your answers to this question constitute the Data Availability Statement and **will be published in the article**, if accepted.<br><br>**Important:** Stating 'data available on request from the author' is not sufficient. If your data are only available upon request, select 'No' for the first question and explain your exceptional situation in the text box.<br><br>Do the authors confirm that all data underlying the findings described in their manuscript are fully available without restriction?	
**Describe where the data may be found in full sentences. If you are copying our sample text, replace any instances of XXX with the appropriate details.**<br><br>• If the data are **held or will be held in a public repository**, include URLs, accession numbers or DOIs. If this information will only be available after acceptance, indicate this by ticking the box below. For example: *All XXX files are available from the XXX database (accession number(s) XXX, XXX.).*<br>• If the data are all contained **within the manuscript and/or Supporting Information files**, enter the following: *All relevant data are within the manuscript and its Supporting Information files.*<br>• If neither of these applies but you are able to provide **details of access elsewhere**, with or without limitations, please do so. For example:<br><br>*Data cannot be shared publicly because of [XXX]. Data are available from the XXX Institutional Data Access / Ethics Committee (contact via XXX) for researchers who meet the criteria for access to confidential data.*<br><br>*The data underlying the results presented in the study are available from (include the name of the third party*	All relevant data are within the manuscript and its Supporting Information files.

and contact information or URL).<br>• This text is appropriate if the data are owned by a third party and authors do not have permission to share the data.<br><br>* typeset	
Additional data availability information:	

Powered by Editorial Manager® and ProduXion Manager® from Aries Systems Corporation

Reporting of research priorities in migraine clinical practice guidelines still need improvement — A methodological analysis

Xuejian Yin[a], Yingdi Liao[b], Zhuo Ye[a], Yiyun Zhao[a], Shiyu An[a], Riai Tang[c], Qinyan Li[c], Shuting Yang[c], Yuze Sun[a], Yicheng Gao[a*], Kai Yuan[a*]

[a]*Second Clinical Medical College, Yunnan University of Chinese Medicine, kunming, China*

[b]*First Clinical Medical College, Yunnan University of Chinese Medicine, kunming, China*

[c]*School of Basic Medical Sciences, Yunnan University of Chinese Medicine, kunming, China*

Co-first authors: Xuejian Yin and Yingdi Liao

[*] Corresponding author: Yicheng Gao and Professor Kai Yuan

Tel. : +0086-0871-65918153

[*] Corresponding author at: 1076 Yu Hua Road, Chenggong District, Kunming, 650500, China

E-mail addresses:

gaoyicheng@ynucm.edu.cn

yk_ynutcm@163.com

ABSTRACT

Objectives: To systematically analyze the content and format of research priorities in clinical practice guidelines (CPGs) for migraine, providing a methodological references for future guideline developers and researchers.

Methods: This methodological systematic analysis involved searching six databases (Embase, PubMed CNKI, VIP, CBM and Wanfang) and the official websites of 39 authoritative organizations, including headache societies, neurology associations, and guideline development bodies. Two reviewers independently performed data retrieval, screening, and extraction. Descriptive statistics were used to calculate the absolute frequencies and proportions of research-focused items. Registered at INPLASY (INPLASY202430055).

Results: Out of 96 included CPGs, 248 research priorities were identified. Only half (n = 48, 50%) of the CPGs explicitly reported research priorities, and only 13.5%(n = 13) included a dedicated section. The primary rationale for proposing these priorities was a lack of sufficient evidence (n = 108, 43.5%). The nomenclature for research priorities was inconsistent, and structured reporting was found to be inadequate. Furthermore, 56% of priorities (n = 139) of priorities focused on the effectiveness of interventions, primarily concerning calcitonin gene-related peptide (CGRP) monoclonal antibodies (n = 20, 8.1%), angiotensin-converting enzyme inhibitors (ACEIs) or angiotensin II receptor blockers (ARBs) (n = 18, 7.3%), and antiepileptic drugs (n = 17, 6.9%).

Conclusions: The reporting of research priorities in migraine CPGs—both in content and format—requires significant improvement. Future efforts should prioritize the

systematic identification and standardized reporting of research gaps in migraine and other disease areas.

Keywords**:** *Research priorities; Migraine; Clinical decision-making; Clinical practice guidelines; Recommendations*

Introduction

Migraine is a prevalent neurological disorder affecting 14% of the global population, characterized by paroxysmal headaches. It is the second leading cause of global disability, imposing a heavy burden on the global healthcare system[1-2]. While numerous clinical practice guidelines (CPGs) have been developed worldwide[3-7], many recommendations within these frameworks lack robust evidence-based support. This evidentiary gap results in significant ambiguity, presenting a formidable challenge for clinical researchers and methodological experts alike.

In the development of CPGs, some recommendations lack sufficient evidence or are based on low-quality evidence, undermining their credibility[8-10]. Therefore, many CPGs highlight areas with weak evidence and establish research priorities for specific sections. The Evidence to Decision (EtD) framework, issued by the Grading of Recommendations Assessment, Development and Evaluation (GRADE) Working Group, explicitly states that research priorities should be integrated into the formulation of CPG recommendations and consistently reported[11-12].—guideline development groups are best positioned to undertake this task[13-15]. This approach not only directs researchers to prioritize critical research areas but also helps them avoid redundant studies in well-established fields, thus preventing the waste of resources[16].

Strictly speaking, research priorities should be evidence-based, but they should not rely solely on evidence. Similar to the formulation of recommendations, they must consider multiple factors[17-21]—often more comprehensive than those for formulating clinical recommendations. For instance, factors related to research resources must also

be considered. Guideline development manuals from various authoritative institutions have outlined the types, content, and format of research priorities[22-24].

Although some studies have explored research priorities, their methodologies have been relatively limited, and the analysis of the reporting format and content has been insufficient[21,25]. This study systematically retrieved and analyzed the content of research recommendations in migraine CPGs, assessed the quality of their reports, and provided valuable references for future migraine research and guideline development.

Methods

Eligibility criteria

This study included all CPGs that provided at least one recommendation for migraine management. Exclusion criteria comprised expert consensus documents, guideline development manuals, duplicate publications, and superseded versions of previously published guidelines.

CPGs sources and searches

XY and YL collaboratively developed the search strategy and independently conducted the search. A total of six databases were searched: Embase, Pubmed, China National Knowledge Infrastructure base (CNKI), Wanfang, Chinese Biomedical Literature Database, and VIP Database for Chinese Technical Periodicals. The keywords used included "Migraine," "Headache," "Migraine with Aura," "evidence-based guideline," "clinical guidance," and "clinical practice guidance," among others. Additionally, we searched the official websites of 39 societies, including the Headache or Migraine Society, the Neurology Society, guideline development organizations, and

authoritative comprehensive societies. The search results were updated prior to submitting the paper. A detailed description of the search strategies can be found in Appendix A.

Definitions

Migraine CPGs were defined as guidelines containing recommendations specifically developed for migraine. Therefore, the CPGs included in this study comprise two categories: (1) Specific migraine CPGs, which focus exclusively on migraine-related recommendations; and (2) Non-specific migraine CPGs, which incorporate migraine-related recommendations as part of a broader clinical scope.

Identification of research priorities

Terms such as "further research," "future research," "limitations," and "recommendations for future research" were used to identify and define research priorities. These terms were commonly found in sections such as the evidence summaries under guideline recommendations, the Discussion, and the Future Research section.

CPGs selection, data extraction and analysis

The CPGs were screened, and data extracted using a two-author back-to-back method, with any discrepancies resolved by a third-author with methodological background (YG). The items of interest in this study were extracted according to a pre-designed data extraction form: (1) basic information related to the CPGs, including publication year, issuing institution, and scope; (2) information related to research priorities, including the number, title, type, rationale, and interventions involved in the

research priorities reports. Notably, we analyzed the structure of the research priorities to determine whether they included any components of the PICO (population, intervention, comparison, and outcome) framework[25]. For data analysis, descriptive statistics were used, calculating the absolute frequency and proportion of the reported items.

Patient and public involvement

No patient involved.

Results

Search results

A total of 29,985 records were retrieved from the database, supplemented by 32 records identified through website searches. After removing duplicates, 20,184 records remained for screening. Initial screening of titles and abstracts resulted in the retention of 114 records for full-text eligibility assessment. Following a detailed review, 18 records were excluded, resulting in a final inclusion of 96 CPGs (Figure 1).

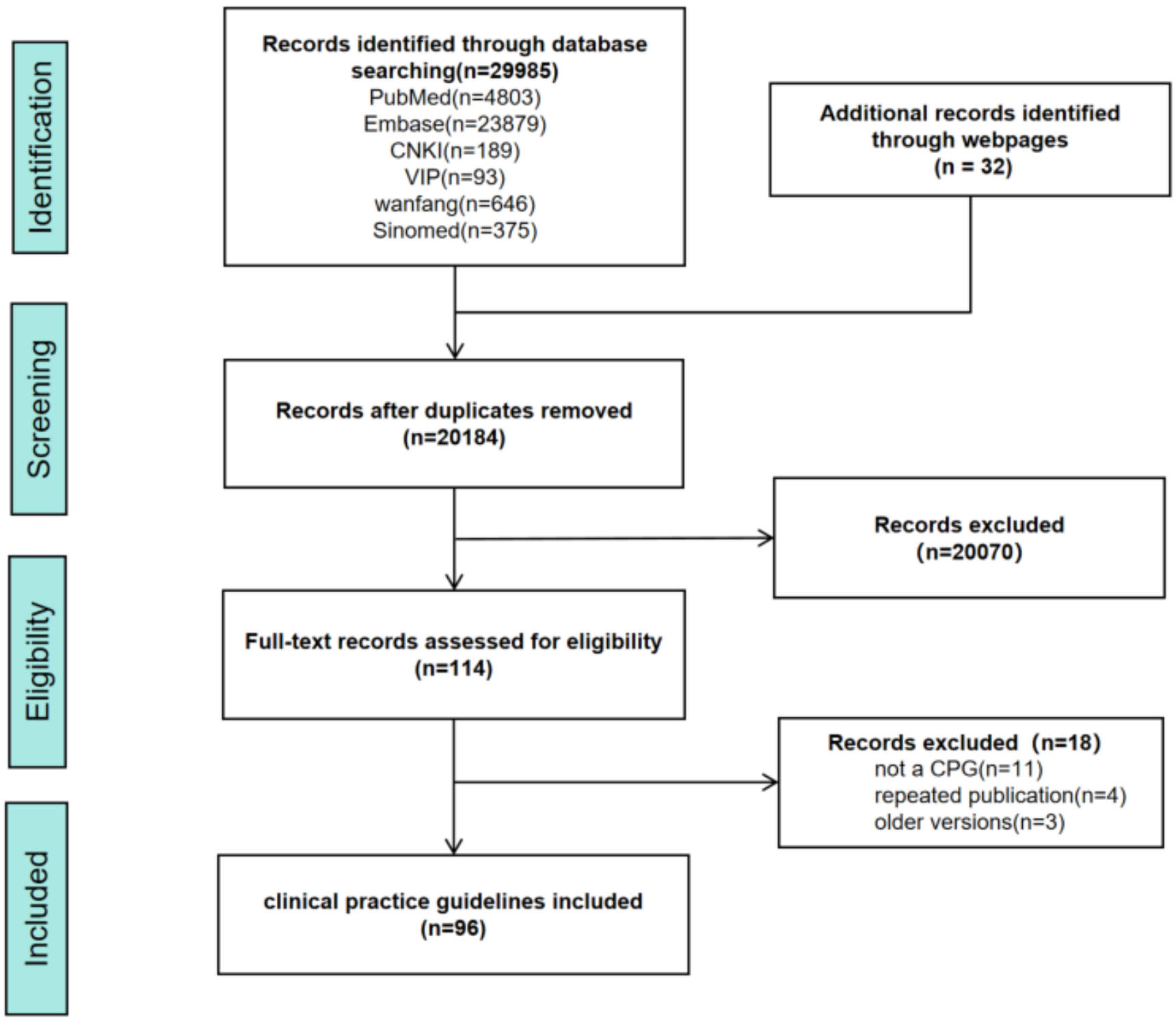

Figure 1. Flowchart of Clinical Practice Guidelines Screening

Guidelines characteristics

Of the 96 included CPGs, 63 (65.6%) were classified as specific migraine guidelines, while 33 (34.4%) were categorized as non-specific. Regarding their primary focus, 70 (72.9%) guidelines centered on disease management, whereas only 26 (27.1%) focused specifically on therapeutic interventions. (Table 1).

Table 1 Characteristics of included clinical practice guidelines（N=96）

Variable	Frequency	Percentage
**Scope of CPGs**		
Treatment	38	39.6
Diagnosis and treatment (or management)	21	21.9
Treatment and prevent (or management)	16	16.7
Prevent	11	11.5
Management	10	10.4
**Type of CPGs**		
specific migraine CPGs	63	65.6
non-specific migraine	33	34.4
**Focus of CPGs**		

Focus on disease	70	72.9
Focus on interventions	26	27.1
**Published year**		
2021-2025	43	44.8
2016-2020	24	25.0
2011-2015	14	14.6
2006-2010	12	12.5
Before 2000	3	3.1
**Publication organization**		
Headache or migraine society	35	36.5
Neurology society	18	18.8
Comprehensive Society or Organization	16	16.7
CPGs development organization	16	16.7
CPGs development Group	6	6.3
Others[a]	5	5.2

[a] such as government agencies or societies unrelated to headaches (e.g., the American College of Obstetricians)
CPG, clinical practice guideline; KOA, knee osteoarthritis.

Basic characteristics of research priorities

Only half of the included guidelines (n= 48, 50%) reported research priorities. Among these, 21 (21.9%) incorporated research priorities within the evidence summaries accompanying the recommendations, while only 13 (13.5%) featured a dedicated section for research priorities. Regarding the volume of identified gaps, approximately one-third (n = 34, 35.4%) of the CPGs listed five or fewer research priorities, whereas only 3 (3.1%) identified more than ten. (Table 2).

Table 2 Basic characteristics of research priorities（N=96）

Categories	Clinical practice guidelines	
	Frequency	Percentage(%)
**Report research priorities**		
Reported	48	50.0
Not reported	48	50.0
**What the migraine research priorities were for**		
Specific recommendation in CPGs	19	19.8
Guidelines as a whole	24	25.0
For the entire guideline and specific recommendations	5	5.2
Not reported	48	50.0
**Location of the research priorities**		

Summary of the evidence under each recommendation	21	21.9
Independent section	13	13.5
Discussions of the CPGs	9	9.4
Independent research recommendations	4	4.2
Others[a]	1	1.0
Not reported	48	50.0
**Number of research priorities**		
≤5	34	35.4
6-10	10	10.4
11-15	1	1.0
15-20	0	0.0
≥21	2	2.1
Not reported	48	50.0

[a] Abstract

CPG, clinical practice guideline; KOA, knee osteoarthritis.

Characteristics of the report on research priorities

A total of 248 migraine-related research priorities were identified, the vast majority of which (n = 239, 96.4%) were not presented as clinical questions. The primary reasons for proposing these research priorities (n = 108, 43.5%) were limited or absent evidence. Regarding the nomenclature of research priorities, the most common format was "Further research/trial/study/finding/attention/confirmation/evidence/data/RCT" (n=71, 28.6%), followed by "Future Research/study/direction/trial" (n = 42, 16.9%) and "Recommendations for research/Research Recommendations" (n = 42, 16.9%). Desirable anticipated effects were the most frequently considered factors in research priorities (n = 130, 52.4%), followed by undesirable anticipated effects (n = 82, 33.1%) and "Only the effects was mentioned" (n = 64, 25.8%). Other factors related to the formation of recommendations, such as health equity (n = 6, 2.4%), values and preferences (n = 6, 2.4%), and feasibility (n = 4, 1.6%), were addressed less frequently. Additionally, approximately half of the research priorities contained PI structural

elements, while the more comprehensive and standardized PIC or PICO structures were inadequately reported, with only 55 (22.2%) and 4 (1.6%) research priorities respectively (Table 3).

Table 3 Characteristics of the content and format of research priorities（N=248）

Categories	Research priorities	
	**Frequency**	**Percentage(%)**
**Presentation forms**		
clinical question	9	3.6
Narrative sentences	239	96.4
**Are there any reasons for research priorities?**		
Yes	166	66.9
No	82	33.1
**Reasons for reporting research priorities**		
Insufficient or absent evidence	108	43.5
Low quality of evidence	35	14.1
Study design flaws	10	4.0
Clinical or research needs	20	8.1
Not reported	82	33.1
**Name of the research priorities**		
Further research/trial/study/finding/attention/confirmation/evidence/data/RCT	71	28.6
Future Research/study/direction/trial	42	16.9
Recommendations for research/research recommendations	42	16.9
Areas of insufficient/no evidence	31	12.5
Evidence gaps/research needs/limitations	26	10.5
Research/Study/trial/RCT/treatment option/clinical practice is needed/required/awaited	24	9.7
Additional/new research/trial/study	13	5.2
Need trial/study/RCT/research	9	3.6
Look forward/carry out/conduct/continue research	8	3.2
Be considered/analysed/proven/confirmed	7	2.8
Others[a]	2	0.8
**Research priorities relevant to influencing factors of recommendations**		
Desirable anticipated effects	130	52.4
Only the effects was mentioned	64	25.8
Undesirable anticipated effects	82	33.1
Resource requirements (cost)	8	3.2
Values and preferences	6	2.4
Health equity	6	2.4
Acceptability	5	2.0

Feasibility	4	1.6
Cost-effectiveness	2	0.8
**The structure of the research priorities**		
PI	114	46.0
PIC	55	22.2
P	33	13.3
PIO	14	5.6
I	12	4.8
Unstructured	11	4.4
PICO	4	1.6
PO	3	1.2
O	2	0.8

[a] The challenge ahead, Studies should try to.

PICO (population, intervention, comparison, and outcome)

Directions and interventions in research priorities

More than half (n = 148, 59.7%) of the migraine research priorities focused on medications, primarily including calcitonin gene-related peptide (CGRP) monoclonal antibodies, angiotensin-converting enzyme inhibitors (ACEIs) or angiotensin II receptor blockers (ARBs) , and antiepileptic drugs， with the proportions being 8.1% (n = 20), 7.3% (n = 18), and 6.9% (n = 17), respectively. The research directions mainly focus on efficacy (n = 139, 56.0%), treatment plan (n = 23, 9.3%), and research methodology issues (n = 19, 7.7%) (Figure 2 and Supplementary Table 1).

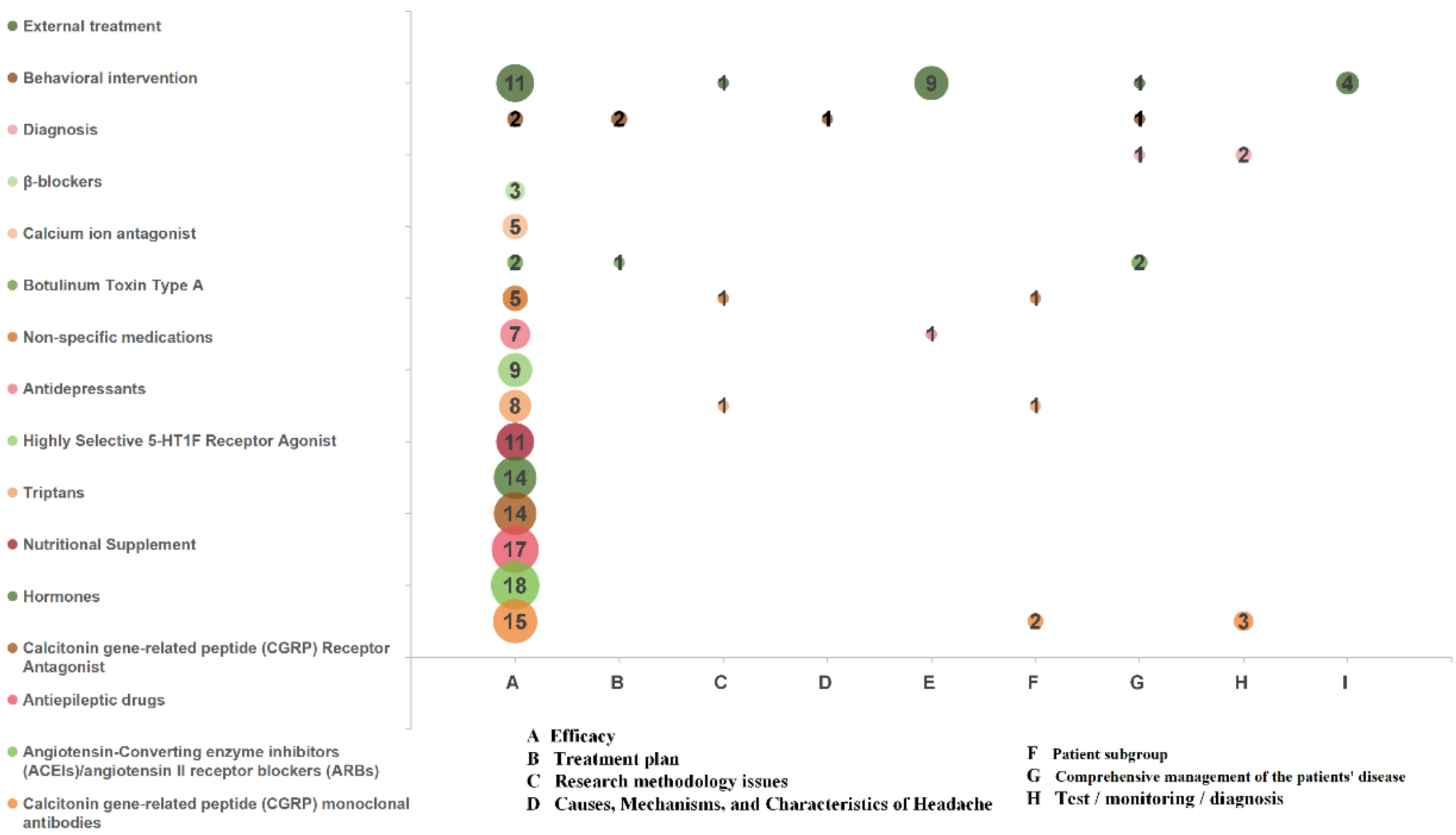

Figure 2 Directions and interventions in research priorities（N=248）

Discussion

Principal findings

This study included 96 CPGs, identifying a total of 248 research priorities. Only half of the CPGs reported research priorities, and only 13 CPGs included a distinct section on research priorities. The primary reason for proposing these priorities (n = 108, 43.5%) was insufficient or absent evidence. The naming conventions varied, and the structured reporting was inadequate. Additionally, the research priorities primarily (n = 139, 56%) focused on the effectiveness of interventions, which mainly involved CGRP monoclonal antibodies (n = 20, 8.1%), ACEIs or ARBs (n = 18, 7.3%), and antiepileptic drugs (n = 17, 6.9%).

Strength and limitations

As far as we know, this study is the first to systematically analyze research priorities in migraine CPGs. Through systematic retrieval, screening, and analysis, we ensured the comprehensive identification of research priorities in these guidelines. We

not only summarized the reported content of migraine research priorities, such as titles, locations, and research directions, but also analyzed their format and the use of the PICO structure, providing reference evidence for future research priority reporting.

However, our study has limitations. We believe that the evidence base for the identified research priorities varies. While weaker evidence typically demands more research, resources are limited and must be prioritized for the most urgent and significant directions. Therefore, we emphasize that weak evidence is neither a prerequisite nor a necessary condition for generating research priorities. Consequently, we did not investigate the specific reasons behind each priority. It is also important to note that different guideline development groups may define the lack of evidence or low-quality evidence differently. We respect the consensus decisions made by these groups and did not further assess their evidence-based judgments.

Relation to previous work

Some studies suggested that research priorities should be structured according to the principles of EPICOT (evidence, population, intervention, comparison, outcome and time), with consideration given to both the type of research and the disease burden[26-28]. A previous study identified gaps in research priorities and evidence, noting that 70% of the research gaps in CPGs issued by Cystic Fibrosis Foundation were not the research priorities identified in the CPGs[29]. Another study examined the content and format of research priorities reported in guidelines for knee osteoarthritis CPGs[30].

Although these studies share similarities with our work, a key difference is how we present research priorities as an independent section in CPGs. Our study further

emphasizes the content and format of research priorities, providing a more accurate reflection of their true essence.

Implications for research and guidelines

During the development of CPGs, the formulation and reporting of research priorities are crucial components that require significant effort. This process not only identifies gaps in existing evidence but also provides key research directions for CPGs users and investigators.

First, CPGs should standardize the positioning and nomenclature of research priorities to facilitate quick identification. The structure of these priorities should be standardized using frameworks such as PICO or EPICOT, which can aid in their development and enable their translation into clinical questions. This approach ensures that the implications of structural elements are maintained between clinical practice and future research, avoiding the need to reconstruct clinical questions. Second, a substantial portion of research recommendations focuses on the efficacy of interventions. Future studies should strengthen the evidence on the efficacy of various migraine treatments, particularly for drugs with high research demand. For interventions with minimal research demand, further exploration into the underlying reasons is needed to determine whether the limited number of research priorities reflects sufficient evidence, low clinical demand, or other factors. It is important to note that the absence or limited number of research priorities in certain areas does not necessarily indicate that the evidence in those fields is robust or of high quality. Third, greater attention should be given to dimensions such as health equity, feasibility, and values

and preferences, with an emphasis on expanding research beyond efficacy. Additionally, methodological research priorities must receive adequate attention and reporting. Finally, areas with substantial existing evidence should be explicitly identified as research priorities to avoid redundant studies.

CONCLUSION

The content (e.g., dimensions considered, research directions) and format (e.g., degree of structuring, name, and location) of research priorities in migraine CPGs still have significant room to improvement. Future efforts should focus on the identification and reporting of research priorities in CPGs for migraine or other diseases.

Declarations

Ethics approval and consent to participate

Not applicable

Consent for publication

Not applicable

Availability of data and materials

All data generated or analysed during this study are included in this published article and its supplementary information files.

Competing interests

All the authors state they have no conflicts of interest with regard to the content of this article.

Patient and public involvement

None.

Funding

This work was supported by Yunnan Province's Xingdian Talents Support Program Young Scholars [grant numbers: XDYC-QNRC-2022-0269]. Funding sources have no involvement in any of the activities conducted in this article.

Data availability

Data is provided within the manuscript or supplementary information files.

Registration

We have registered at INPLASY with the registration number INPLASY202430055.

Authors' contributions

Xuejian Yin: Investigation, Formal analysis, Writing original draft. Yingdi Liao: Extracting data, Formal analysis, Writing original draft- review & editing. Zhuo Ye: Extracting data, Formal analysis, Writing original draft. Yiyun Zhao: Extracting data, Writing - review & editing. Shiyu An: Extracting data, Writing - review & editing. Riai Tang: Formal analysis, Writing - review & editing. Qinyan Li: Formal analysis, Writing - review & editing. Shuting Yang: Extracting data, Writing - review & editing. Yuze Sun: Formal analysis, Writing - review & editing. Yicheng Gao: Conceptualization, Formal analysis, Writing - review & editing. Kai Yuan: Conceptualization, Funding acquisition, Investigation, Formal analysis, Methodology, Project administration.

Acknowledgements

The authors would to thank Jimei Yang from Yunnan University of Chinese Medicine, for contributing to the literature screening, data extraction and visualization.

REFERENCES

1. GBD 2016 Disease and Injury Incidence and Prevalence Collaborators. Global, regional, and national incidence, prevalence, and years lived with disability for 328 diseases and injuries for 195 countries, 1990-2016: a systematic analysis for the global burden of disease study 2016. Lancet. (2017) 390:1211–59.

2. Linde, M, Gustavsson, A, Stovner, LJ, Steiner, TJ, Barré, J, Katsarava, Z, et al. The cost of headache disorders in Europe: the Eurolight project. Eur J Neurol. (2012) 19:703–11.

3. Ornello R, Caponnetto V, Ahmed F, Al-Khazali HM, Ambrosini A, Ashina S, et al. Evidence-based guidelines for the pharmacological treatment of migraine. Cephalalgia. 2025 Apr;45(4):3331024241305381.

4. Qaseem A, Tice JA, Etxeandia-Ikobaltzeta I, Wilt TJ, Harrod CS, Cooney TG, et al. Pharmacologic Treatments of Acute Episodic Migraine Headache in Outpatient Settings: A Clinical Guideline From the American College of Physicians. Ann Intern Med. 2025 Apr;178(4):571-578.

5. Qaseem A, Cooney TG, Etxeandia-Ikobaltzeta I, Wilt TJ, Harrod CS, Tice JA, et al. Prevention of Episodic Migraine Headache Using Pharmacologic Treatments in

Outpatient Settings: A Clinical Guideline From the American College of Physicians. Ann Intern Med. 2025 Mar;178(3):426-433.

6. La Touche R, Fierro-Marrero J, Sánchez-Ruíz I, Rodríguez de Rivera-Romero B, Cabrera-López CD, Lerma-Lara S, et al. Prescription of therapeutic exercise in migraine, an evidence-based clinical practice guideline. J Headache Pain. 2023 Jun 7;24(1):68.
7. Al-Suwaidan FA, Almudaiheem HY, Alotaibi HF, Alhazzani WA, Al-Quliti KW, Alenzi KA, et al. Saudi clinical practice guidelines for the treatment and prevention of migraine headache. Neurosciences (Riyadh). 2025 Apr;30(2):77-91.
8. Qureshi O, Ross JS, Ramachandran R, Habib AR. Level of Evidence and Strength of Recommendations in US Medical Society Clinical Practice Guidelines, 2019-2023: A Cross-Sectional Analysis. J Gen Intern Med. 2025 Dec 22.
9. Institute of Medicine (US) Committee on Standards for Developing Trustworthy Clinical Practice Guidelines, Graham R, Mancher M, Miller Wolman D, Greenfield S, Steinberg E, eds. Clinical Practice Guidelines We Can Trust. Washington (DC): National Academies Press (US); 2011.
10. Neumann I, Santesso N, Akl EA, Rind DM, Vandvik PO, Alonso-Coello P, et al. A guide for health professionals to interpret and use recommendations in guidelines developed with the GRADE approach. J Clin Epidemiol. 2016 Apr;72:45-55.
11. Lotfi T, Hajizadeh A, Moja L, Akl EA, Piggott T, Kredo T, et al. A taxonomy and framework for identifying and developing actionable statements in guidelines suggests avoiding informal recommendations. J Clin Epidemiol. 2022

Jan;141:161-171.

12. Andrews J, Guyatt G, Oxman AD, Alderson P, Dahm P, Falck-Ytter Y, et al. GRADE guidelines: 14. Going from evidence to recommendations: the significance and presentation of recommendations. J Clin Epidemiol. 2013 Jul;66(7):719-25.
13. Moberg J, Oxman AD, Rosenbaum S, Schünemann HJ, Guyatt G, Flottorp S, et al. The GRADE Evidence to Decision (EtD) framework for health system and public health decisions. Health Res Policy Syst. 2018 May 29;16(1):45.
14. Norris SL, Aung MT, Chartres N, Woodruff TJ. Evidence-to-decision frameworks: a review and analysis to inform decision-making for environmental health interventions. Environ Health. 2021 Dec 8;20(1):124.
15. Meneses-Echavez JF, Bidonde J, Yepes-Nuñez JJ, Poklepović Peričić T, Puljak L, Bala MM, et al. Evidence to decision frameworks enabled structured and explicit development of healthcare recommendations. J Clin Epidemiol. 2022 Oct;150:51-62.
16. Scott J, Checketts JX, Horn JG, Cooper C, Vassar M. "Knee osteoarthritis and current research for evidence-are we on the right way?". Int Orthop. 2018 Sep;42(9):2105-2112.
17. Ruxrungtham K. Guidelines to improve urticarial care and the remaining research gaps. Asian Pac J Allergy Immunol. 2016 Sep;34(3):179-180.
18. Singh Ospina N, Rodriguez-Gutierrez R, Brito JP, Young WF Jr, Montori VM. Is the endocrine research pipeline broken? A systematic evaluation of the Endocrine

Society clinical practice guidelines and trial registration. BMC Med. 2015 Aug 12;13:187.

19. Chou R, Ballantyne JC, Fanciullo GJ, Fine PG, Miaskowski C. Research gaps on use of opioids for chronic noncancer pain: findings from a review of the evidence for an American Pain Society and American Academy of Pain Medicine clinical practice guideline. J Pain. 2009 Feb;10(2):147-59.

20. Zhang W, Moskowitz RW, Nuki G, Abramson S, Altman RD, Arden N, et al. OARSI recommendations for the management of hip and knee osteoarthritis, part I: critical appraisal of existing treatment guidelines and systematic review of current research evidence. Osteoarthritis Cartilage. 2007 Sep;15(9):981-1000.

21. Research gaps from evidence-based contraception guidance: the US Medical Eligibility Criteria for Contraceptive Use, 2016, and the US Selected Practice Recommendations for Contraceptive Use, 2016

22. World Health Organization.WHO handbook for guideline development[M].2nd ed.Vienna:World Health Organization,2014.

23. National Health and Medical Research Council.Procedures and requirements for meeting the 2011 NHMRC standard for clinical practice guidelines-Summary for developers[M].Melbourne:National Health and Medical Research Council,2011.

24. Scottish Intercollegiate Guidelines Network.A guideline developer's handbook[M].Edinburgh:Scottish Intercollegiate Guidelines Network,2019.

25. Ruxrungtham K. Guidelines to improve urticarial care and the remaining research gaps. Asian Pac J Allergy Immunol. 2016 Sep;34(3):179-180.

26. Brown P, Brunnhuber K, Chalkidou K, Chalmers I, Clarke M, Fenton M, et al. How to formulate research recommendations. BMJ. 2006 Oct 14;333(7572):804-6.

27. Vlassov V. How to formulate research recommendations: format is not enough. BMJ 2006;333:917.

28. Greenhalgh T. How to formulate research recommendations: the pie or the slice? BMJ 2006;333:917.

29. Robinson KA, Saldanha IJ, McKoy NA. Identification of research gaps from evidence-based guidelines: a pilot study in cystic fibrosis. Int J Technol Assess Health Care 2011;27:247–52.

30. Gao Y, Liu Z, Cao R, Feng Y, Tao L, Su C, et al. Reporting form and content of research priorities identified in knee osteoarthritis clinical practice guidelines: a methodological literature analysis. BMJ Open. 2024 Apr 10;14(4):e076107.

Click here to access/download
Supporting Information
PRISMA_2020_checklist.docx

Click here to access/download
Supporting Information - Compressed/ZIP File Archive
supplementary files.zip
