# Supplementary material for: Reporting of research priorities in migraine clinical practice guidelines still need improvement—A methodological analysis
Source: PLoS One. 2026 Oct 30;21(10):e0361302. doi: 10.1371/journal.pone.0361302 (PMC13722188; doi:10.1371/journal.pone.0361302)
Supplement: Supplementary file 2 [file pone.0361302.s005.pdf]

General comments

This manuscript examines research priorities in clinical practice guidelines (CPG) related to migraine treatment. This topic is interesting and potentially useful, as CPGs can provide a structured overview of the research gaps identified during guideline development. The manuscript appears thorough and systematic in identifying and describing the overall themes of the CPGs, with clearly defined criteria and methods.

Overall, I think this is a useful and interesting contribution to the literature which evaluates themes in the reporting of research gaps and priorities. Given the substantial efforts directed to developing frameworks for systematic and evidence-based review, there is value in assessing whether these approaches are being used in practice. The manuscript highlights an opportunity to improve consistency and utility of CPGs. However, I think the manuscript would benefit from major revisions to strengthen the framing and provide more context and details for the reader. This could also increase the usefulness of the manuscript for a broader audience.

Specific comments

- The manuscript would benefit from a clearer discussion about the primary role of clinical practice guidelines, which to inform clinicians and decision-makers about treatment options in the context of evidence-based care. The identification of suggested research opportunities is a valuable secondary function of the guideline development process, as the expert panels are well-placed to identify research gaps. The authors should consider reframing the introduction and discussion to reflect this distinction. The inconsistent reporting identified could be presented constructively as an opportunity to improve if barriers were overcome.
- Greater clarity on the distinction between research gaps, needs and priorities would be beneficial. Alternatively, the authors could make more explicit how these terms are defined and applied in the manuscript. There is some discussion about the role of factors, such as resources, in the decision to undertake research (para starting line 85). However, this could include some specific examples of research gaps vs. research priorities.
- In the abstract, it is not clear what the CPGs are being compared against to be considered 'inadequate' and 'inconsistent'; this could be specified.
- It would be useful to have more details about the filtering of ~20,000 records down to 114. This would improve transparency and reproducibility.
- Figure 2 is difficult to interpret given all the different coloured dots. A table or alternative presentation method may be more useful. Another option might be to line up the figure legend with the 'dots'. A figure caption should also be provided.
- The authors may wish to consider possible reasons for the inconsistent reporting of research priorities. For example, guideline development methods and

frameworks have been refined over time. This may provide some context for the older guidelines that do not provide detailed research recommendations.

- Section starting line 224 – this section is somewhat general and would benefit from revision. It could be improved by stating how the knee osteoporosis and cystic fibrosis studies compare more specifically to this migraine study.
- If guidelines were translated, I suggest that a comment is added to describe the translation (e.g. I was only able to find Selbstmedikation bei Migräne und beim Kopfschmerz vom Spannungstyp written in German).
- The Data availability statement is 'No – some restrictions will apply', but the authors also confirm that all relevant data is included. Something to clarify.

Minor corrections

- Abstract - The INPLASY code is incorrect as this code and study appears to be published already. The correct code appears to be: INPLASY202630045
- Extra space in line 78
- Line 81 – stray dash
- Line 110 – suggest referring to Appendix B.
- Line 112 – suggest adding a specific timeframe e.g. Month/Year to save the reader looking up the submission date.
- Line 135 – is this the correct reference (#25?). This appears to be a duplicate reference.
- KOA (knee osteoarthritis) in captions for Tables 1 or 2 – this abbreviation does not appear to be used in these tables.
- Extra space in line 192
- Line 263 – 'significant room for improvement' rather than 'significant room to improvement'
- Appendix B – suggest that the Chinese text in the column labelled 'type of arthritis' is translated to English (appears to be Specific/Non-specific migraine guidelines), as well as the heading of that column.
- Appendix B – is reference 75 meant to be highlighted?
