# Supplementary material for: Reporting of research priorities in migraine clinical practice guidelines still need improvement—A methodological analysis
Source: PLoS One. 2026 Oct 30;21(10):e0361302. doi: 10.1371/journal.pone.0361302 (PMC13722188; doi:10.1371/journal.pone.0361302)
Supplement: Supplementary file 3 [file pone.0361302.s001.docx]

Supplementary Table 1 Search Strategy

| **Database** | **Step** | **Search Strategy** | **References** |
| --- | --- | --- | --- |
| **PubMed** | **#1** | "Migraine Disorders"[MeSH Terms] OR "Migraine without Aura"[MeSH Terms] OR "Migraine with Aura"[MeSH Terms] OR "Headache"[MeSH Terms] OR "migraine"[Title/Abstract] OR "Migraine Disorders"[Title/Abstract] OR "headache*"[Title/Abstract] OR "head ache*"[Title/Abstract] OR "migrain*"[Title/Abstract] OR "cephalgi*"[Title/Abstract] OR "cephalalgi*"[Title/Abstract] OR "hemicrani*"[Title/Abstract] OR "Headache Disorders"[Title/Abstract] OR "Headache"[Title/Abstract] | [154999](https://pubmed.ncbi.nlm.nih.gov/?term="Migraine+Disorders"[MeSH+Terms]+OR+"Migraine+without+Aura"[MeSH+Terms]+OR+"Migraine+with+Aura"[MeSH+Terms]+OR+"Headache"[MeSH+Terms]+OR+"migraine"[Title/Abstract]+OR+"Migraine+Disorders"[Title/Abstract]+OR+"headache*"[Title/Abstract]+OR+"head+ache*"[Title/Abstract]+OR+"migrain*"[Title/Abstract]+OR+"cephalgi*"[Title/Abstract]+OR+"cephalalgi*"[Title/Abstract]+OR+"hemicrani*"[Title/Abstract]+OR+"Headache+Disorders"[Title/Abstract]+OR+"Headache"[Title/Abstract]&sort=pubdate&size=200&ac=no) |
|  | **#2** | "Practice Guideline"[Publication Type] OR "Guideline"[Publication Type] OR "Guidelines as Topic"[MeSH Terms] OR "clinical guidance"[Title/Abstract] OR "clinical practice guidance"[Title/Abstract] OR "guideline*"[Title/Abstract] OR "guidance*"[Title/Abstract] OR "clinical guidance"[Title/Abstract] OR "clinical guideline"[Title/Abstract] OR "evidence-based guideline"[Title/Abstract] | 901246 |
|  | #3 | #1 AND #2 | [4803](https://pubmed.ncbi.nlm.nih.gov/?term=("Migraine+Disorders"[MeSH+Terms]+OR+"Migraine+without+Aura"[MeSH+Terms]+OR+"Migraine+with+Aura"[MeSH+Terms]+OR+"Headache"[MeSH+Terms]+OR+"migraine"[Title/Abstract]+OR+"Migraine+Disorders"[Title/Abstract]+OR+"headache*"[Title/Abstract]+OR+"head+ache*"[Title/Abstract]+OR+"migrain*"[Title/Abstract]+OR+"cephalgi*"[Title/Abstract]+OR+"cephalalgi*"[Title/Abstract]+OR+"hemicrani*"[Title/Abstract]+OR+"Headache+Disorders"[Title/Abstract]+OR+"Headache"[Title/Abstract])+AND+("Practice+Guideline"[Publication+Type]+OR+"Guideline"[Publication+Type]+OR+"Guidelines+as+Topic"[MeSH+Terms]+OR+"clinical+guidance"[Title/Abstract]+OR+"clinical+practice+guidance"[Title/Abstract]+OR+"guideline*"[Title/Abstract]+OR+"guidance*"[Title/Abstract]+OR+"clinical+guidance"[Title/Abstract]+OR+"clinical+guideline"[Title/Abstract]+OR+"evidence-based+guideline"[Title/Abstract])&sort=pubdate&size=200&ac=no) |
| **Embase** | #1 | migraine/exp OR migraine:ti,ab,kw OR 'migraine disorders':ti,ab,kw OR 'migraine without aura':ti,ab,kw OR headache*:ti,ab,kw OR 'head ache*':ti,ab,kw OR migrain*:ti,ab,kw OR cephalgi*:ti,ab,kw OR hemicrani*:ti,ab,kw OR 'headache disorders'/exp OR headache:ti,ab,kw | 482328 |
|  | #2 | ('practice guideline'/exp OR 'clinical practice guidance':ti,ab,kw OR 'guideline*':ti,ab,kw OR 'guidance*':ti,ab,kw OR 'clinical guidance':ti,ab,kw OR 'clinical guideline':ti,ab,kw OR 'evidence-based guideline':ti,ab,kw) | 1656863 |
|  | #3 | #1 AND #2 | 23879 |
| **China National Knowledge Infrastructure** | #1 | (SU %='偏头痛' + '头痛') | 57980 |
|  | #2 | (SU%= '指南' + '临床指南' + '临床实践指南' + '循证指南' + '循证临床指南'+ '循证临床实践指南' + '诊疗方案' + '诊疗规范' + '诊疗指南') | 120584 |
|  | #3 | #1 AND #2 | 189 |
| **VIP Database for Chinese Technical Periodicals** | #1 | (M=(偏头痛 OR 头痛)) | 34866 |
|  | #2 | (M=(指南 OR 临床指南 OR 临床实践指南 OR 循证指南 OR 循证临床指南 OR 循证临床实践指南 OR 诊疗指南 OR 诊疗方案 OR 诊疗规范)) | 97357 |
|  | #3 | #1 AND #2 | 93 |
| **Wanfang** | #1 | 主题:("偏头痛"or"头痛") | 126239 |
|  | #2 | 主题:("指南"or"临床指南"or"临床实践指南"or"循证指南"or"循证临床指南"or"循证临床实践指南"or"诊疗指南"or"诊疗规范"or"诊疗方案") | 1094148 |
|  | #3 | #1 AND #2 | 646 |
| **Chinese Biomedical Literature Database** | #1 | "偏头痛"[常用字段:智能] OR "头痛"[常用字段:智能] | 222544 |
|  | #2 | ("指南"[常用字段:智能] OR "临床指南"[常用字段:智能] OR "临床实践指南"[常用字段:智能] OR "循证指南"[常用字段:智能] OR "循证临床指南"[常用字段:智能] OR "循证临床实践指南"[常用字段:智能] OR "诊疗指南"[常用字段:智能] OR "诊疗规范"[常用字段:智能] OR "诊疗方案"[常用字段:智能]) | 91025 |
|  | #3 | #1 AND #2 | 375 |

**website search**

| **Number** | **Institutions** | **Webpages** |
| --- | --- | --- |
| 1 | American Headache Society(AHS) | https://americanheadachesociety.org/ |
| 2 | American College of Physicians(ACH) | https://www.acponline.org/ |
| 3 | American Academy of Neurology(AAN) | https://www.aan.com/ |
| 4 | American College of Physicians(ACP) | https://www.acponline.org |
| 5 | The British Association for the Study of Headache (**BASH**) | https://bash.org.uk/ |
| 6 | Canadian Medical Association（CMA） | http://www.cma.ca/ |
| 7 | Center　for　Health　Evidence(CHE) | http://www.cche.net/ |
| 8 | [The Canadian Headache Society](https://www.headachesociety.ca/)(**CHS**) | https://[www.headachesociety.ca](https://www.headachesociety.ca/) |
| 9 | [Canadian Migraine Society](https://www.migrainesociety.ca/)(**CMS**) | https://www.migrainesociety.ca/ |
| 10 | Canadian Association of Emergency Physician(**CAEP**) | https://caep.ca/ |
| 11 | China Association of Chinese Medicine(**CACM**) | https://www.cacm.org.cn/ |
| 12 | China Medical Doctor Association(**CMDA**) | https://www.cmda.net/ |
| 13 | Chinese Association of Integrative Medicine(**CAIM**) | http://www.caim.org.cn/ |
| 14 | The [Canadian Task Force on Preventive Health Care](https://www.canada.ca/en/public-health/services/chronic-diseases/canadian-task-force-on-preventive-health-care.html)(CTFPHC) | https://canadiantaskforce.ca/ |
| 15 | Centers for Disease Control and Prevention(CDC) | https://www.cdc.gov/ |
| 16 | Danish Headache Society(**DHS**) | https://dhos.dk/ |
| 17 | European Academy of Neurology(EAN) | https://[www.ean.org](https://www.bing.com/alink/link?url=http://www.ean.org/&source=serp-local&h=mdhgy4WiD0N4G2w/5bnCNtfhjRuAQVDGg2llQO5XbpI=&p=lw_magsmlt&ig=6D8DDD93C8C64F9692A6E89F30095F14&ypid=YN9002x8113190542848645230) |
| 18 | European Headache Federation(EHF) | https://www.ehf-headache.com/ |
| 19 | French Society of Neurology(SFN) | https://www.sf-neuro.org/ |
| 20 | International Headache Society （IHC） | https://ihs-headache.org/en/ |
| 21 | Federatie Medisch Specialisten(FMS) | https://demedischspecialist.nl/over-ons |
| 22 | Spanish Society of Neurology(SEN) | https://www.sen.es/ |
| 23 | The Swiss Headache Society (SHS) | https://www.headache.ch/ |
| 24 | Headache Society of Singapore(HSS) | https://headachesocietysingapore.com/ |
| 25 | Medlive | https://www.medlive.cn/ |
| 26 | Société Française d'Etude des Migraines et Céphalées(SFEMC) | https://sfemc.fr/ |
| 27 | The Japanese Headache Society(JHC) | https://www.jhsnet.net/english/english_about.html |
| 28 | The Taiwan Headache Society(THC) | https://taiwanheadache.org.tw |
| 29 | The U.S. Preventive Services Task Force | [https://www.uspreventiveservicestaskforce.org](https://www.uspreventiveservicestaskforce.org/uspstf/home) |
| 30 | Scottish Intercollegiate Guidelines Network(SIGN) | https://www.sign.ac.uk |
| 31 | Guidelines　International　Network(GIN) | http://www.g-i-n.net/ |
| 32 | The National Institute for Health and Care Excellence(NICE) | http://www.nice.org.uk/ |
| 33 | The Royal Australian College of General Practitioners (RACGP) | https://www.racgp.org.au/ |
| 34 | Australian Government National Healthand Medical Research Council(NHMRC) | https://www.nhmrc.gov.au/ |
| 35 | The New Zealand Ministry of Health | http://www.health.govt.nz/ |
| 36 | World Health Organization(WHO) | http://www.who.int/ |
| 37 | Agency for Health care Research and Quality(AHRQ) | https://www.ahrq.gov/ |
| 38 | Guidelines　Advisory　Committee(GAC) | http://www.gacguidelines.ca/ |
| 39 | Ministry of Health Malaysia(MaHTAS) | http://www.moh.gov.my/ |
