# Supplementary material for: Reporting of research priorities in migraine clinical practice guidelines still need improvement—A methodological analysis
Source: PLoS One. 2026 Oct 30;21(10):e0361302. doi: 10.1371/journal.pone.0361302 (PMC13722188; doi:10.1371/journal.pone.0361302)
Supplement: Supplementary file 4 [file pone.0361302.s002.docx]

**Supplementary Table 2 Included clinical practice guidelines**

| **Number** | **Title of clinical practice guideline** | **Year** | **Type of migraine guidelines** |
| --- | --- | --- | --- |
| 1 | European Headache Federation guideline on the use of monoclonal antibodies targeting the calcitonin gene related peptide pathway for migraine prevention – 2022 update | 2022 | Specific migraine CPGs |
| 2 | Comprehensive management of adults with chronic migraine: Clinical practice guidelines in Mexico | 2021 | Specific migraine CPGs |
| 3 | Clinical Practice Guideline for Headache Disorders 2021 | 2025 | Non-specific migraine CPGs |
| 4 | Fremanezumab for preventing migraine | 2022 | Specific migraine CPGs |
| 5 | Transcutaneous electrical stimulation of the supraorbital nerve for treating and preventing migraine | 2022 | Specific migraine CPGs |
| 6 | Treatment of migraine attacks and preventive treatment of migraine | 2022 | Specific migraine CPGs |
| 7 | Rimegepant for treating migraine | 2023 | Specific migraine CPGs |
| 8 | Rimegepant for preventing migraine | 2023 | Specific migraine CPGs |
| 9 | Eptinezumab for preventing migraine | 2023 | Specific migraine CPGs |
| 10 | 2022 Taiwan Guidelines for Acute Treatment of Migraine | 2022 | Specific migraine CPGs |
| 11 | Prescription of therapeutic exercise in migraine, an evidence-based clinical practice guideline | 2023 | Specific migraine CPGs |
| 12 | Pharmacological management of migraine | 2023 | Specific migraine CPGs |
| 13 | Atogepant for preventing migraine | 2024 | Specific migraine CPGs |
| 14 | International Headache Society practice recommendations for the acute pharmacological treatment of migraine | 2024 | Specific migraine CPGs |
| 15 | CROATIAN GUIDELINES FOR SPECIFIC PREVENTIVE TREATMENT OF MIGRAINE WITH MONOCLONAL ANTIBODIES TARGETING CALCITONIN GENE-RELATED PEPTIDE (CGRP) (EPT INEZUMAB, FREMANEZUMAB,AND GALCANEZUMAB) OR THE CGRP RECEPTOR (ERENUMAB) | 2024 | Specific migraine CPGs |
| 16 | Prevention of Episodic Migraine Headache Using Pharmacologic Treatments in Outpatient Settings: A Clinical Guideline From the American College of Physicians | 2025 | Specific migraine CPGs |
| 17 | Practice advisory for intravenous management of headache disorders in hospitalized patients: a review of the evidence and consensus recommendations | 2025 | Non-specific migraine CPGs |
| 18 | Saudi clinical practice guidelines for the treatment and prevention of migraine headache | 2025 | Specific migraine CPGs |
| 19 | Pharmacologic Treatments of Acute Episodic Migraine Headache in Outpatient Settings: A Clinical Guideline From the American College of Physicians | 2025 | Specific migraine CPGs |
| 20 | Evidence-based guidelines for the pharmacological treatment of migraine | 2025 | Specific migraine CPGs |
| 21 | Galcanezumab for preventing migraine | 2020 | Specific migraine CPGs |
| 22 | Standard headache and neuralgia treatments and SARS-CoV-2: opinion of the Spanish Society of Neurology’s Headache Study GroupTratamientos habituales utilizados en cefaleas, neuralgias y SARS-CoV-2. Posicionamiento del grupo de estudio de cefaleas de la Sociedad Española de Neurología | 2020 | Non-specific migraine CPGs |
| 23 | MANUAL de Práctica Clínica en CEFALEAS：Recomendaciones Diagnóstico-Terapéuticas de la Sociedad Española de Neurología 2020 | 2020 | Non-specific migraine CPGs |
| 24 | Practice guideline update: Pharmacologic treatment for pediatric migraine prevention. | 2019 | Specific migraine CPGs |
| 25 | Guideline on the use of onabotulinumtoxinA in chronic migraine: a consensus statement from the European Headache Federation | 2018 | Specific migraine CPGs |
| 26 | Medicamenteuze behandeling migraine en MOH | 2018 | Specific migraine CPGs |
| 27 | Practice guideline update: Acute treatment of migraine in children and adolescents | 2018 | Specific migraine CPGs |
| 28 | Medical Treatment Guidelines for Preventive Treatment of Migraine | 2017 | Specific migraine CPGs |
| 29 | Erenumab for preventing migraine | 2021 | Specific migraine CPGs |
| 30 | Reference programme: diagnosis and treatment of headache disorders and facial pain. Danish Headache Society, 3rd edition, 2020 | 2020 | Non-specific migraine CPGs |
| 31 | Headache Treatment Guidelines of the Portuguese Headache Society – 2021 | 2021 | Non-specific migraine CPGs |
| 32 | Neuromodulación en cefaleas y neuralgias craneofaciales: Guía de la Sociedad Espanola ˜ de Neurología y de la Sociedad Espanola ˜ de Neurocirugía | 2021 | Non-specific migraine CPGs |
| 33 | Migraine treatment recommendations developed by an Expert Group of the Polish Headache Society, the Headache Section of the Polish Neurological Society, and the Polish Pain Society | 2021 | Specific migraine CPGs |
| 34 | Management of Adults With Acute Migraine in the Emergency Department: The American Headache Society Evidence Assessment of Parenteral Pharmacotherapies | 2016 | Specific migraine CPGs |
| 35 | Transcutaneous stimulation of the cervical branch of the vagus nerve for cluster headache and migraine | 2016 | Non-specific migraine CPGs |
| 36 | Consensus Guide on Pericranial Nerve Block Anesthesia Techniques | 2012 | Non-specific migraine CPGs |
| 37 | Hormonal contraceptives and risk of ischemic stroke in women with migraine: a consensus statement from the European Headache Federation (EHF) and the European Society of Contraception and Reproductive Health (ESC) | 2017 | Specific migraine CPGs |
| 38 | Medical Treatment Guidelines for Acute Migraine Attacks | 2017 | Specific migraine CPGs |
| 39 | Occipital nerve stimulation for intractable chronic migraine | 2013 | Specific migraine CPGs |
| 40 | Canadian Headache Society Guideline Acute Drug Therapy for Migraine Headache | 2013 | Specific migraine CPGs |
| 41 | Transcranial magnetic stimulation for treating and preventing migraine | 2014 | Specific migraine CPGs |
| 42 | Canadian Headache Society systematic review and recommendations on the treatment of migraine pain in emergency settings | 2014 | Specific migraine CPGs |
| 43 | European headache federation guideline on the use of monoclonal antibodies acting on the calcitonin gene related peptide or its receptor for migraine prevention | 2019 | Specific migraine CPGs |
| 44 | Consensus of the Brazilian Headache Society on the treatment of chronic migraine | 2019 | Specific migraine CPGs |
| 45 | Therapy recommendations for primary headaches | 2019 | Non-specific migraine CPGs |
| 46 | Effect of exogenous estrogens and progestogens on the course of migraine during reproductive age: a consensus statement by the European Headache Federation (EHF) and the European Society of Contraception and Reproductive Health(ESCRH) | 2018 | Specific migraine CPGs |
| 47 | Recommendations for the management of a headache in an emergency | 2018 | Non-specific migraine CPGs |
| 48 | Clinical practice guideline for chronic headache 2013 | 2019 | Non-specific migraine CPGs |
| 49 | Revised guidelines of the French Headache Society for the diagnosis and management of migraine in adults. Part 1: Diagnosis and assessment | 2021 | Specific migraine CPGs |
| 50 | Headache: pregnancy and breastfeeding. Recommendations of the Spanish Society of Neurology’s Headache Study Group | 2022 | Non-specific migraine CPGs |
| 51 | Headaches in Pregnancy and Postpartum: ACOG Clinical Practice Guideline No. 3 | 2022 | Non-specific migraine CPGs |
| 52 | Approach to headache disorders and the management of migraine: consensus guidelines from the Headache Society of Singapore, first edition (2023) | 2023 | Specific migraine CPGs |
| 53 | Guidelines for the diagnosis and management of migraine in clinical practice | 1997 | Specific migraine CPGs |
| 54 | EFNS guidelines on the molecular diagnosis of channelopathies, epilepsies, migraine, stroke, and dementias | 2010 | Non-specific migraine CPGs |
| 55 | Percutaneous closure of patent foramen ovale for recurrent migraine | 2010 | Specific migraine CPGs |
| 56 | Therapie der Migräne | 2012 | Specific migraine CPGs |
| 57 | International standard of Traditional Chinese Medicine Techniques: Traditional Chinese Medicine guidelines for acute primary headache (2022) | 2022 | Non-specific migraine CPGs |
| 58 | Guidance to the blood-letting puncture and cupping in the treatment of migraine | 2023 | Specific migraine CPGs |
| 59 | Guía práctica diagnóstico terapéutica de la Cefalea del adulto y el niño en Urgencias | 2016 | Non-specific migraine CPGs |
| 60 | Guidance for the management of headache in sport on behalf of The Royal College of General Practitioners and The British Association for the Study of Headache | 2010 | Non-specific migraine CPGs |
| 61 | Guidelines for the diagnosis and management of migraine in adults and children | 2012 | Specific migraine CPGs |
| 62 | Italian guidelines for primary headaches: 2012 revised version | 2012 | Non-specific migraine CPGs |
| 63 | TCM Diagnostic and Treatment Guidelines for Pediatric Migraine | 2023 | Non-specific migraine CPGs |
| 64 | Practice Parameter: Pharmacological treatment of migraine headache in children and adolescents | 2011 | Specific migraine CPGs |
| 65 | Guidelines for All Healthcare Professionals in the Diagnosis and Management of Migraine Tension-Type Headache Cluster Headache Medication-Overuse Headache | 2010 | Non-specific migraine CPGs |
| 66 | The Use of NaoAn Dropping Pills/Capsules in the Treatment of Migraine: Expert Consensus | 2024 | Specific migraine CPGs |
| 67 | Princípios europeus da abordagem das cefaleias comuns nos cuidados de saúde primários | 2010 | Non-specific migraine CPGs |
| 68 | Clinical practice guideline for migraine with traditional Chinese medicine( draft version for comments) | 2020 | Specific migraine CPGs |
| 69 | Canadian Headache Society Guideline for Migraine Prophylaxis | 2012 | Specific migraine CPGs |
| 70 | Botulinum toxin type A for the prevention of headaches in adults with chronic migraine | 2012 | Specific migraine CPGs |
| 71 | Headaches in over 12s: diagnosis and management | 2025 | Non-specific migraine CPGs |
| 72 | Health Care Guideline: Diagnosis and Treatment of Headache | 2011 | Non-specific migraine CPGs |
| 73 | Diagnosis and Treatment of Migraine with the Integrated Traditional Chinese and Western Medicine | 2022 | Specific migraine CPGs |
| 74 | Guideline for the Diagnosis and Treatment of Chinese Medicine in Vestibular Migraine | 2024 | Specific migraine CPGs |
| 75 | Headache | 2017 | Non-specific migraine CPGs |
| 76 | Standardized Management of Primary Headaches in Adults: Recommendations from the Pan Yangtze River Delta Expert Group for Diagnosis and Treatment in Headache (2024) | 2024 | Non-specific migraine CPGs |
| 77 | Chinese practice guidelines for diagnosis and treatment of migraine (1st edition, Chinese Society of Neurology) | 2023 | Specific migraine CPGs |
| 78 | 中国偏头痛中西医结合防治指南（2022年） | 2022 | Specific migraine CPGs |
| 79 | Guidelines for the nonpharmacologic management of migraine in clinical practice | 1998 | Specific migraine CPGs |
| 80 | 中国偏头痛急性期治疗指南（第一版） | 2022 | Specific migraine CPGs |
| 81 | 中国偏头痛防治指南 | 2016 | Specific migraine CPGs |
| 82 | Migraine treatment in developmental age: guidelines update | 2010 | Specific migraine CPGs |
| 83 | Behandlung der Migräne und idio pathischer Kopfschmerzsyndrome in Schwangerschaft und Stillzeit | 2009 | Non-specific migraine CPGs |
| 84 | RECOMENDAÇÕES TERAPÊUTICAS PARA CEFALEIAS | 2009 | Non-specific migraine CPGs |
| 85 | 中国偏头痛诊治指南 | 2022 | Specific migraine CPGs |
| 86 | 中国偏头痛诊断治疗指南 | 2011 | Specific migraine CPGs |
| 87 | EFNS guideline on the drug treatment of migraine – revised report of an EFNS task force | 2009 | Specific migraine CPGs |
| 88 | Selbstmedikation bei Migräne und beim Kopfschmerz vom Spannungstyp | 2009 | Non-specific migraine CPGs |
| 89 | Diagnosis and management of headache in adults:A national clinical guideline | 2008 | Non-specific migraine CPGs |
| 90 | Therapie primärer chronischer Kopfschmerzen: Chronische Migräne, chronischer Kopfschmerz vom Spannungstyp und andere chronische tägliche Kopfschmerzen. | 2007 | Non-specific migraine CPGs |
| 91 | Canadian Association of Emergency Physicians Guidelines for the acute management of migraine headache | 1999 | Specific migraine CPGs |
| 92 | Update: pharmacologic treatment for episodic migraine prevention in adults | 2012 | Specific migraine CPGs |
| 93 | International Headache Society Global Practice Recommendations for Preventive Pharmacological Treatment of Migraine | 2024 | Specific migraine CPGs |
| 94 | Entspannungsverfahren und verhaltenstherapeutische Interventionen zur Behandlung der Migräne | 2017 | Specific migraine CPGs |
| 95 | The Primary Care Management of Headache: Synopsis of the 2020 U.S. Department of Veterans Affairs and U.S. Department of Defense Clinical Practice Guideline | 2022 | Non-specific migraine CPGs |
| 96 | 2023 U.S. Department of Veterans Affairs and U.S. Department of Defense Clinical Practice Guideline for the Management of Headache | 2023 | Non-specific migraine CPGs |

The guideline used original titles, for example, guideline available only in German, the titles were presented solely in German.
