# Supplementary material for: Reporting of research priorities in migraine clinical practice guidelines still need improvement—A methodological analysis
Source: PLoS One. 2026 Oct 30;21(10):e0361302. doi: 10.1371/journal.pone.0361302 (PMC13722188; doi:10.1371/journal.pone.0361302)
Supplement: Supplementary file 5 [file pone.0361302.s003.docx]

**Supplementary Table 3 Directions and interventions in research priorities（N=248）**

| **Intervention** | **Frequency** | **Percentage(%)** |
| --- | --- | --- |
| Medicine | 148 | 59.7 |
| External treatment | 26 | 10.5 |
| Behavioral intervention | 6 | 2.4 |
| Diagnosis | 3 | 1.2 |
| Other interventions | 4 | 1.6 |
| Not involved | 64 | 25.8 |
| **The categories of drugs involved** |  |  |
| No specific drug name | 29 | 11.7 |
| Calcitonin gene-related peptide (CGRP) monoclonal antibodies | 20 | 8.1 |
| Angiotensin-Converting enzyme inhibitors (ACEIs)/angiotensin II receptor blockers (ARBs) | 18 | 7.3 |
| Antiepileptic drugs | 17 | 6.9 |
| Calcitonin gene-related peptide (CGRP) Receptor Antagonist | 14 | 5.6 |
| Hormones | 14 | 5.6 |
| - Nutritional Supplement | 11 | 4.4 |
| Triptans | 10 | 4.0 |
| Highly Selective 5-HT1F Receptor Agonist | 9 | 3.6 |
| Antidepressants | 8 | 3.2 |
| Non-specific medications. | 7 | 2.8 |
| Botulinum Toxin Type A | 6 | 2.4 |
| Calcium ion antagonist | 5 | 2.0 |
| β-blockers | 3 | 1.2 |
| Other Pharmaceuticals | 3 | 1.2 |
| **Research directions** |  |  |
| Efficacy | 139 | 56.0 |
| Treatment plan | 23 | 9.3 |
| Research methodology issues | 19 | 7.7 |
| Causes, Mechanisms, and Characteristics of Headache | 16 | 6.5 |
| Implementation details and outcomes | 16 | 6.5 |
| Patient subgroup | 14 | 5.6 |
| Comprehensive management of the patients' disease | 10 | 4.0 |
| Test / monitoring / diagnosis | 6 | 2.4 |
| Follow-up or prognosis | 5 | 2.0 |
