# Supplementary material for: Reporting of research priorities in migraine clinical practice guidelines still need improvement—A methodological analysis
Source: PLoS One. 2026 Oct 30;21(10):e0361302. doi: 10.1371/journal.pone.0361302 (PMC13722188; doi:10.1371/journal.pone.0361302)
Supplement: Peer review history

# Peer review: Decision Letter 0

**DOI:** 10.1371/journal.pone.0361302.r001

**Authors:** Sercan Ergün

1 Jul 2026

Dear Dr. Gao,

Thank you for submitting your manuscript to PLOS ONE. After careful consideration, we feel that it has merit but does not fully meet PLOS ONE’s publication criteria as it currently stands. Therefore, we invite you to submit a revised version of the manuscript that addresses the points raised during the review process.

==============================

**ACADEMIC EDITOR:**  Based on the reviewers' evaluations, I have decided that the manuscript should be revised before it can be considered further.

plosone@plos.org. When you're ready to submit your revision, log on to https://www.editorialmanager.com/pone/ and select the 'Submissions Needing Revision' folder to locate your manuscript file.

- A letter that responds to each point raised by the academic editor and reviewer(s). You should upload this letter as a separate file labeled 'Response to Reviewers'.
- A marked-up copy of your manuscript that highlights changes made to the original version. You should upload this as a separate file labeled 'Revised Manuscript with Track Changes'.
- An unmarked version of your revised paper without tracked changes. You should upload this as a separate file labeled 'Manuscript'.

If you would like to make changes to your financial disclosure, please include your updated statement in your cover letter. Guidelines for resubmitting your figure files are available below the reviewer comments at the end of this letter.

If applicable, we recommend that you deposit your laboratory protocols in protocols.io to enhance the reproducibility of your results. Protocols.io assigns your protocol its own identifier (DOI) so that it can be cited independently in the future. For instructions see: https://journals.plos.org/plosone/s/submission-guidelines#loc-laboratory-protocols. Additionally, PLOS ONE offers an option for publishing peer-reviewed Lab Protocol articles, which describe protocols hosted on protocols.io. Read more information on sharing protocols at https://plos.org/protocols?utm_medium=editorial-email&utm_source=authorletters&utm_campaign=protocols.

As the corresponding author, your ORCID iD is verified in the submission system and will appear in the published article. PLOS supports the use of ORCID, and we encourage all coauthors to register for an ORCID iD and use it as well. Please encourage your coauthors to verify their ORCID iD within the submission system before final acceptance, as unverified ORCID iDs will not appear in the published article. *Only*  the individual author can complete the verification step; PLOS staff *cannot*  verify ORCID iDs on behalf of authors.

We look forward to receiving your revised manuscript.

Kind regards,

Sercan Ergün

Academic Editor

PLOS One

Journal Requirements:

When submitting your revision, we need you to address these additional requirements.

1. Please ensure that your manuscript meets PLOS ONE's style requirements, including those for file naming. The PLOS ONE style templates can be found at https://journals.plos.org/plosone/s/file?id=wjVg/PLOSOne_formatting_sample_main_body.pdf and https://journals.plos.org/plosone/s/file?id=ba62/PLOSOne_formatting_sample_title_authors_affiliations.pdf

2. Thank you for stating in your Funding Statement:

[This work was supported by Yunnan Province's Xingdian Talents Support Program Young Scholars [grant numbers: XDYC-QNRC-2022-0269]. Funding sources have no involvement in any of the activities conducted in this article.].

Please provide an amended statement that declares *all* the funding or sources of support (whether external or internal to your organization) received during this study, as detailed online in our guide for authors at http://journals.plos.org/plosone/s/submit-now.  Please also include the statement “There was no additional external funding received for this study.” in your updated Funding Statement.

Please include your amended Funding Statement within your cover letter. We will change the online submission form on your behalf.

3. Please remove any funding-related text from the manuscript. Please note that funding information should not appear in any section or other areas of your manuscript. We will publish the funding statement that is present in the Financial Disclosure section of the online submission form.

4. We note that you have indicated that there are restrictions to data sharing for this study. PLOS only allows data to be available upon request if there are legal or ethical restrictions on sharing data publicly. For more information on unacceptable data access restrictions, please see http://journals.plos.org/plosone/s/data-availability#loc-unacceptable-data-access-restrictions.

Before we proceed with your manuscript, please address the following prompts:

a) If there are ethical or legal restrictions on sharing a de-identified data set, please explain them in detail (e.g., data contain potentially identifying or sensitive patient information, data are owned by a third-party organization, etc.) and who has imposed them (e.g., a Research Ethics Committee or Institutional Review Board, etc.). Please also provide contact information for a data access committee, ethics committee, or other institutional body to which data requests may be sent.

b) If there are no restrictions, please upload the minimal anonymized data set necessary to replicate your study findings to a stable, public repository and provide us with the relevant URLs, DOIs, or accession numbers. Please see http://www.bmj.com/content/340/bmj.c181.long for guidelines on how to de-identify and prepare clinical data for publication. For a list of recommended repositories, please see https://journals.plos.org/plosone/s/recommended-repositories. You also have the option of uploading the data as Supporting Information files, but we would recommend depositing data directly to a data repository if possible.

Please update your Data Availability statement in the submission form accordingly.

5. PLOS requires an ORCID iD for the corresponding author in Editorial Manager on papers submitted after December 6th, 2016. Please ensure that you have an ORCID iD and that it is validated in Editorial Manager. To do this, go to ‘Update my Information’ (in the upper left-hand corner of the main menu), and click on the Fetch/Validate link next to the ORCID field. This will take you to the ORCID site and allow you to create a new iD or authenticate a pre-existing iD in Editorial Manager.

6. Please include captions for your Supporting Information files at the end of your manuscript, and update any in-text citations to match accordingly. Please see our Supporting Information guidelines for more information: http://journals.plos.org/plosone/s/supporting-information.

7. Thank you for providing your underlying data as Supporting Information.

We note that the data set contains text or data that is not in English. Please note that PLOS is an English-language publisher, so we require data sets to be provided in English as well. Please upload an English-language version of your data set.

This will also allow us to determine if your data follows PLOS standards per our Data Availability policy here: https://journals.plos.org/plosone/s/data-availability

If the reviewer comments include a recommendation to cite specific previously published works, please review and evaluate these publications to determine whether they are relevant and should be cited. There is no requirement to cite these works unless the editor has indicated otherwise.

Additional Editor Comments:

Based on the reviewers' evaluations, I have decided that the manuscript should be revised before it can be considered further.

[Note: HTML markup is below. Please do not edit.]

Reviewers' comments:

Reviewer's Responses to Questions

**Comments to the Author**

1. Is the manuscript technically sound, and do the data support the conclusions?

Reviewer #1: Yes

Reviewer #2: Yes

Reviewer #3: No

Reviewer #4: Yes

**********

2. Has the statistical analysis been performed appropriately and rigorously?

Reviewer #1: I Don't Know

Reviewer #2: Yes

Reviewer #3: I Don't Know

Reviewer #4: N/A

**********

3. Have the authors made all data underlying the findings in their manuscript fully available?

The PLOS Data policy

Reviewer #1: Yes

Reviewer #2: Yes

Reviewer #3: Yes

Reviewer #4: Yes

**********

4. Is the manuscript presented in an intelligible fashion and written in standard English?

Reviewer #1: Yes

Reviewer #2: Yes

Reviewer #3: Yes

Reviewer #4: Yes

**********

Reviewer #1: No comments to explain, the analysis is well applied, the results well shown and the importance explained

No comments to explain, the analysis is well applied, the results well shown and the importance explained

Reviewer #2: Firstly this is a very well written study in clear scientific English. Presumably it is based on a large database, and is very statistical attempting to analyze the criteria included, and ones that should be concluded in migraine guidelines. I doubt there is any other study not mentioned to compare it with. It should help guide authors preparing new guidelines.

Reviewer #3: This manuscript is on review of published articles .The authors could adopt a proper process like systematic review for presenting the findings and giving the recommendations. The purpose , objectives and outcome of this review are not given clearly. The entire manuscript may be made more clear. In the present form the manuscript is not giving a value addition to the existing body of knowledge.

Reviewer #4: Please find comments attached and pasted below.

General comments

This manuscript examines research priorities in clinical practice guidelines (CPG) related to migraine treatment. This topic is interesting and potentially useful, as CPGs can provide a structured overview of the research gaps identified during guideline development. The manuscript appears thorough and systematic in identifying and describing the overall themes of the CPGs, with clearly defined criteria and methods.

Overall, I think this is a useful and interesting contribution to the literature which evaluates themes in the reporting of research gaps and priorities. Given the substantial efforts directed to developing frameworks for systematic and evidence-based review, there is value in assessing whether these approaches are being used in practice. The manuscript highlights an opportunity to improve consistency and utility of CPGs. However, I think the manuscript would benefit from major revisions to strengthen the framing and provide more context and details for the reader. This could also increase the usefulness of the manuscript for a broader audience.

Specific comments

• The manuscript would benefit from a clearer discussion about the primary role of clinical practice guidelines, which to inform clinicians and decision-makers about treatment options in the context of evidence-based care. The identification of suggested research opportunities is a valuable secondary function of the guideline development process, as the expert panels are well-placed to identify research gaps. The authors should consider reframing the introduction and discussion to reflect this distinction. The inconsistent reporting identified could be presented constructively as an opportunity to improve if barriers were overcome.

• Greater clarity on the distinction between research gaps, needs and priorities would be beneficial. Alternatively, the authors could make more explicit how these terms are defined and applied in the manuscript. There is some discussion about the role of factors, such as resources, in the decision to undertake research (para starting line 85). However, this could include some specific examples of research gaps vs. research priorities.

• In the abstract, it is not clear what the CPGs are being compared against to be considered ‘inadequate’ and ‘inconsistent’; this could be specified.

• It would be useful to have more details about the filtering of ~20,000 records down to 114. This would improve transparency and reproducibility.

• Figure 2 is difficult to interpret given all the different coloured dots. A table or alternative presentation method may be more useful. Another option might be to line up the figure legend with the ‘dots’. A figure caption should also be provided.

• The authors may wish to consider possible reasons for the inconsistent reporting of research priorities. For example, guideline development methods and frameworks have been refined over time. This may provide some context for the older guidelines that do not provide detailed research recommendations.

• Section starting line 224 – this section is somewhat general and would benefit from revision. It could be improved by stating how the knee osteoporosis and cystic fibrosis studies compare more specifically to this migraine study.

• If guidelines were translated, I suggest that a comment is added to describe the translation (e.g. I was only able to find Selbstmedikation bei Migräne und beim Kopfschmerz vom Spannungstyp written in German).

• The Data availability statement is ‘No – some restrictions will apply’, but the authors also confirm that all relevant data is included. Something to clarify.

Minor corrections

- Abstract - The INPLASY code is incorrect as this code and study appears to be published already. The correct code appears to be: INPLASY202630045

- Extra space in line 78

- Line 81 – stray dash

- Line 110 – suggest referring to Appendix B.

- Line 112 – suggest adding a specific timeframe e.g. Month/Year to save the reader looking up the submission date.

- Line 135 – is this the correct reference (#25?). This appears to be a duplicate reference.

- KOA (knee osteoarthritis) in captions for Tables 1 or 2 – this abbreviation does not appear to be used in these tables.

- Extra space in line 192

- Line 263 – ‘significant room for improvement’ rather than ‘significant room to improvement’

- Appendix B – suggest that the Chinese text in the column labelled ‘type of arthritis’ is translated to English (appears to be Specific/Non-specific migraine guidelines), as well as the heading of that column.

- Appendix B – is reference 75 meant to be highlighted?

**********

what does this mean?). If published, this will include your full peer review and any attached files.

If you choose “no”, your identity will remain anonymous but your review may still be made public.

**Do you want your identity to be public for this peer review?**  For information about this choice, including consent withdrawal, please see our Privacy Policy

Reviewer #1: **Yes:**  Rita Abdo

Reviewer #2: **Yes:**  Jeremy Hornibrook

Reviewer #3: No

Reviewer #4: No

**********

[NOTE: If reviewer comments were submitted as an attachment file, they will be attached to this email and accessible via the submission site. Please log into your account, locate the manuscript record, and check for the action link "View Attachments". If this link does not appear, there are no attachment files.]

To ensure your figures meet our technical requirements, please review our figure guidelines: https://journals.plos.org/plosone/s/figures

You may also use PLOS’s free figure tool, NAAS, to help you prepare publication quality figures: https://journals.plos.org/plosone/s/figures#loc-tools-for-figure-preparation.

NAAS will assess whether your figures meet our technical requirements by comparing each figure against our figure specifications.

Attachment Submitted filename: PONE-D-26-15753.pdf

Attachment Submitted filename: review.pdf

# Author comment: Author response to Decision Letter 1

**DOI:** 10.1371/journal.pone.0361302.r002

31 Jul 2026

Point-by-point response to reviewers

Dear Editors and Reviewers,

Thank you for your letter and for the reviewers’ comments for our manuscript entitled “Reporting of research priorities in migraine clinical practice guidelines still need improvement — A methodological analysis”.(ID: PONE-D-26-15753). Those comments are all valuable and very helpful for revising and improving our paper. Revised words are marked in red in the paper. The point to point corrections in the paper and the responds to the reviewer’s comments are as following:

Response to the reviewer’s comments:

General comments:

This manuscript examines research priorities in clinical practice guidelines (CPG) related to migraine treatment. This topic is interesting and potentially useful, as CPGs can provide a structured overview of the research gaps identified during guideline development. The manuscript appears thorough and systematic in identifying and describing the overall themes of the CPGs, with clearly defined criteria and methods.

Overall, I think this is a useful and interesting contribution to the literature which evaluates themes in the reporting of research gaps and priorities. Given the substantial efforts directed to developing frameworks for systematic and evidence-based review, there is value in assessing whether these approaches are being used in practice. The manuscript highlights an opportunity to improve consistency and utility of CPGs. However, I think the manuscript would benefit from major revisions to strengthen the framing and provide more context and details for the reader. This could also increase the usefulness of the manuscript for a broader audience.

Response: Thank you for your positive comments. Your comments have given us great confidence and encouraged us to engage in further research in this field.

Specific comments:

1.The manuscript would benefit from a clearer discussion about the primary role of clinical practice guidelines, which to inform clinicians and decision-makers about treatment options in the context of evidence-based care. The identification of suggested research opportunities is a valuable secondary function of the guideline development process, as the expert panels are wellplaced to identify research gaps. The authors should consider reframing the introduction and discussion to reflect this distinction. The inconsistent reporting identified could be presented constructively as an opportunity to improve if barriers were overcome.

Response: We appreciate this insightful comment. We fully acknowledge that the primary function of clinical practice guidelines is to inform clinicians and healthcare decision-makers of treatment options under the framework of evidence-based care, whereas identifying research gaps is a valuable secondary output of guideline development, given expert panels’ unique advantages in spotting unaddressed research needs. Accordingly, we have restructured relevant paragraphs in the Introduction and Discussion to clearly articulate this distinction.

We have added the following statements to the Introduction section (Line 83-89): “Clinical practice guidelines are principally developed to assist clinicians and healthcare decision-makers in selecting appropriate therapeutic strategies based on available evidence. While identifying unanswered clinical questions is not their core objective, guideline expert panels are uniquely positioned to recognize gaps in existing evidence.14-16 Therefore, systematically extracting research priorities from migraine guidelines constitutes a valuable secondary application of guideline documents”. Additionally, we have added the following statements to the Discussion section (Line 283-289) : “It should be emphasized that determining research priorities should not divert the core responsibility of guideline developement, which is to provide guidance for clinicians and decision-makers; whereas identifying research priorities remains a secondary objective within the guideline development. By adopting a unified reporting framework, insights from expert panels on unresolved migraine-related questions can be systematically documented, ensuring the optimization of future research directions and priorities.”

2.Greater clarity on the distinction between research gaps, needs and priorities would be beneficial. Alternatively, the authors could make more explicit how these terms are defined and applied in the manuscript. There is some discussion about the role of factors, such as resources, in the decision to undertake research (para starting line 85). However, this could include some specific examples of research gaps vs. research priorities.

Response: We greatly appreciate your constructive suggestion on distinguishing the concepts of research gaps, research needs and research priorities, which helps clarify the core logic of this study and resolve potential conceptual ambiguities for readers. Distinctions between research gaps, research needs and research priorities are elaborated as follows:

�Research gaps refer to the mismatch between clinical questions in urgent need of evidentiary support and the body of existing research evidence. Objectively, they reflect inadequate, low-quality or entirely absent evidence available to address clinical questions.

�Research needs denote clinical or disciplinary demands arising from unresolved research gaps. Such demands only mirror the objective requirement for supplementary evidence, without comprehensive appraisal of implementation prerequisites including research funding, patient accessibility and technical feasibility.

�Research priorities represent the screening and ranking of research needs via multidimensional comprehensive trade-offs, encompassing evidentiary sufficiency, clinical disease burden, research resource availability, health equity and other dimensions. This prioritized research agenda constitutes a subjectively ranked scheme formulated for existing research gaps and needs following systematic evaluation of multiple influencing factors.

It should be clarified that the above three terms carry largely consistent core implications across all clinical practice guidelines included in this studyy, with only minor differences in phrasing. To address this conceptual ambiguity raised in your comment, we have added the following statement to the revised manuscript (Line 135-137): “It should be emphasized that the above may involve slight differences in meaning, yet they share a consistent fundamental direction; therefore, we have all categorized them as research priorities.”

Additionally, we have provided supplementary explanations regarding the influencing factors involved in conducting the study and included illustrative examples (Line 93-98): “For instance, factors related to research resources must also be considered. Additional factors related to research resources and research equity must also be considered. For instance, an intervention may require substantial resources to demonstrate its efficacy but yield benefits only for a very small population, resulting in more disadvantages than advantages at the research, thereby diminishing its research priority.”

We sincerely hope that the revisions addressed above are satisfactory to you.

3. In the abstract, it is not clear what the CPGs are being compared against to be considered ‘inadequate’ and ‘inconsistent’; this could be specified.

Response: We appreciate this valuable comment. The improvements addressed specific inadequate and inconsistent, primarily those related to the low reporting rates of relevant entries or the varied titles used for research priorities. Due to space limitations in the abstracts, detailed explanations were not feasible. We revised the wording by replacing (Line 37-38) "The nomenclature for research priorities was inconsistent" with "The nomenclature for research priorities varies widely" and added the phrase (Line 30-31) "Extract information such as the name, type, and quantity of research priorities" in the methods section to enhance clarity for readers.

4. It would be useful to have more details about the filtering of ~20,000 records down to 114. This would improve transparency and reproducibility.

Response: Thank the reviewer for the comments. The researches' search results section strictly followed the literature screening procedures outlined in the systematic review. To enhance clarity, we replaced (Line 156-157) "Initial screening of titles and abstracts resulted in the retention of 114 records for full-text eligibility assessment." with "Following screening of titles and abstracts, 20,070 records were excluded, leaving 114 records for full-text eligibility assessment." We synchronized and modified the corresponding text in the picture.

5. Figure 2 is difficult to interpret given all the different coloured dots. A table or alternative presentation method may be more useful. Another option might be to line up the figure legend with the ‘dots’. A figure caption should also be provided.

Response: We would like to express our sincere gratitude to the reviewers for their valuable suggestions regarding Figure 2. First, we have revised the legend to align it with the positions of the colored dots, thereby facilitating reader comprehension; second, we have added a complete standardized caption below Figure 2: "The X-axis represents research directions, while the Y-axis lists various migraine intervention strategies."

6. The authors may wish to consider possible reasons for the inconsistent reporting of research priorities. For example, guideline development methods and frameworks have been refined over time. This may provide some context for the older guidelines that do not provide detailed research recommendations.

Response: We are grateful to the reviewer for recommendation. We have added a section in the discussion that addresses how the updated guideline development methodology and framework impact the guidelines (Line 279-283): “Regarding the methodology for guideline development, developers should recognize that guideline-specific methodologies are continuously evolving; therefore, the latest development approaches (such as GRADE approach) must be adopted to prevent diminished credibility and clinical relevance of guidelines due to methodological shortcomings.”

7. Section starting line 224 – this section is somewhat general and would benefit from revision. It could be improved by stating how the knee osteoporosis and cystic fibrosis studies compare more specifically to this migraine study.

Response: Thanks a lot the reviewer for the suggestion. We agree and changed (Line 240-243) "A previous study identified gaps in research priorities and evidence, noting that 70% of the research gaps in CPGs issued by Cystic Fibrosis Foundation were not the research priorities identified in the CPGs" to "A previous study identified gaps in research priorities and evidence, noting that 70% of the research gaps in CPGs issued by Cystic Fibrosis Foundation were not explicitly listed as research priorities within these guidelines but rather stemmed purely from insufficient evidence supporting the formal recommendations." in the text. Meanwhile, we changed (Line 246-254) "Although these studies share similarities with our work, a key difference is how we present research priorities as an independent section in CPGs. Our study further emphasizes the content and format of research priorities, providing a more accurate reflection of their true essence" to "While these prior studies align with our research focus on research priorities, they fundamentally differ from this study either by failing to treat the research priorities as independent and components of guideline reports or by addressing diseases distinct from those examined here. Through analysis across various diseases, this study further clarifies the content and format of research priorities, providing a more accurate representation of their true essence. By simultaneously examining both the structured reporting standards for research priorities and their substantive thematic characteristics, our analysis advances the field and offers a more detailed and comprehensive understanding of their core attributes".

8. If guidelines were translated, I suggest that a comment is added to describe the translation (e.g. I was only able to find Selbstmedikation bei Migräne und beim Kopfschmerz vom Spannungstyp written in German).

Response: We are grateful to the reviewer for the valuable comments. We want to do an explanation here. For guidelines requiring translation, we have retained the original titles in the attachments. For instance, guidelines available only in German were labeled solely with their German titles to distinguish them from those published in English. Additionally, annotations have been added beneath the tables in Appendix B to enhance clarity for readers.

9. The Data availability statement is ‘No – some restrictions will apply’, but the authors also confirm that all relevant data is included. Something to clarify.

Response: We thank the reviewer for pointing out this conflicting expression. We have completely removed the ambiguous phrase from the submission form, and uniformly adopted the standardized data availability statement in the Declarations section of the manuscript: “All data generated or analysed during this study are included in this published article and its supplementary information files”.

Minor corrections:

1. Abstract - The INPLASY code is incorrect as this code and study appears to be published already. The correct code appears to be: INPLASY202630045 .

Response: We are grateful to the reviewer for pointing this out. We re-examined and verified the registration information, updating the correct registration number in the main text to INPLASY2025120098.

2. Extra space in line 78.

Response: We thank the reviewer for this careful check. We have deleted the extra space in the revised manuscript, and all related formatting errors have been corrected.

3. Line 81 – stray dash.

Response: We thank the reviewer for providing valuable feedback. The stray dash has been removed in the revised manuscript.

4. Line 110 – suggest referring to Appendix B.

Response: We appreciate this careful reminder. We have added the citation to Appendix B accordingly in the revised manuscript (Line 159).

5. Line 112 – suggest adding a specific timeframe e.g. Month/Year to save the reader looking up the submission date.

Response: We are grateful to the reviewer for making us aware of the lack of clarity here. We have added the exact date and revised the wording from "The search results were updated prior to submitting the paper" to "The search results have been updated to March 20, 2026, before submitting the paper" (Line 121-122).

6. Line 135 – is this the correct reference (#25?). This appears to be a duplicate reference.

Response: We are very grateful to the reviewer for careful reading. We have re-checked the references and confirmed that this was a duplicate reference. We have updated reference 25 to “Schiavenato M, Chu F. PICO: What it is and what it is not. Nurse Educ Pract. 2021;56:103194” accordingly.

7. KOA (knee osteoarthritis) in captions for Tables 1 or 2 – this abbreviation does not appear to be used in these tables.

Response: Thanks for your careful checks. We are sorry for our carelessness. Based on your comments, we have made the corrections to ensure accuracy in the presentation of your data.

8. Extra space in line 192.

Response: We thank the reviewer for this careful check. We have deleted the extra space in the revised manuscript, and all related formatting errors have been corrected.

9. Line 263 – ‘significant room for improvement’ rather than ‘significant room to improvement’

Response: We were sorry for our mistakes. Thanks for pointing this out. We have revised the incorrect phrase "significant room to improvement" to "significant room for improvement" in the revised manuscript.

10. Appendix B – suggest that the Chinese text in the column labelled ‘type of arthritis’ is translated to English (appears to be Specific/Non-specific migraine guidelines), as well as the heading of that column.

Response: We are deeply grateful for this valuable suggestion. We have translated the Chinese text in Appendix B into English and updated the title of this section to "Type of migraine guidelines".

11. Appendix B – is reference 75 meant to be highlighted?

Response: We thank the reviewer for noticing this issue. The highlighting of reference 75 in Appendix B was an unintended formatting error with no special implication. We have removed the highlight mark for this reference in the revised manuscript.

Attachment Submitted filename: Point-by-point response to reviewers.docx

# Peer review: Decision Letter 1

**DOI:** 10.1371/journal.pone.0361302.r003

**Authors:** Omid Beiki

9 Oct 2026

Reporting of research priorities in migraine clinical practice guidelines still need improvement — A methodological analysis

PONE-D-26-15753R1

Dear Dr. Gao,

We’re pleased to inform you that your manuscript has been judged scientifically suitable for publication and will be formally accepted for publication once it meets all outstanding technical requirements.

Within one week, you’ll receive an e-mail detailing the required amendments. When these have been addressed, you’ll receive a formal acceptance letter and your manuscript will be scheduled for publication.

An invoice will be generated when your article is formally accepted. Please note, if your institution has a publishing partnership with PLOS and your article meets the relevant criteria, all or part of your publication costs will be covered. Please make sure your user information is up-to-date by logging into Editorial Manager at Editorial Manager® and clicking the ‘Update My Information' link at the top of the page. For questions related to billing, please contact billing support.

If your institution or institutions have a press office, please notify them about your upcoming paper to help maximize its impact. If they’ll be preparing press materials, please inform our press team as soon as possible -- no later than 48 hours after receiving the formal acceptance. Your manuscript will remain under strict press embargo until 2 pm Eastern Time on the date of publication. For more information, please contact onepress@plos.org.

Kind regards,

Omid Beiki, MD, PhD, MSc

Academic Editor

PLOS One

Additional Editor Comments (optional):

Reviewers' comments:

Reviewer's Responses to Questions

**Comments to the Author**

Reviewer #4: All comments have been addressed

**********

2. Is the manuscript technically sound, and do the data support the conclusions?

Reviewer #4: Yes

**********

3. Has the statistical analysis been performed appropriately and rigorously?

Reviewer #4: Yes

**********

4. Have the authors made all data underlying the findings in their manuscript fully available?

The PLOS Data policy

Reviewer #4: Yes

**********

5. Is the manuscript presented in an intelligible fashion and written in standard English?

Reviewer #4: Yes

**********

Reviewer #4: Thanks for your responses and for addressing the feedback. Hopefully this has clarified the manuscript for the readers.

**********

what does this mean?). If published, this will include your full peer review and any attached files.

If you choose “no”, your identity will remain anonymous but your review may still be made public.

**Do you want your identity to be public for this peer review?**  For information about this choice, including consent withdrawal, please see our Privacy Policy

Reviewer #4: No

**********

# Editor report: Acceptance letter

**DOI:** 10.1371/journal.pone.0361302.r004

**Authors:** Omid Beiki

PONE-D-26-15753R1

PLOS One

Dear Dr. Gao,

I'm pleased to inform you that your manuscript has been deemed suitable for publication in PLOS One. Congratulations! Your manuscript is now being handed over to our production team.

At this stage, our production department will prepare your paper for publication. This includes ensuring the following:

* All references, tables, and figures are properly cited

* All relevant supporting information is included in the manuscript submission,

* There are no issues that prevent the paper from being properly typeset

You will receive further instructions from the production team, including instructions on how to review your proof when it is ready. Please keep in mind that we are working through a large volume of accepted articles, so please give us a few days to review your paper and let you know the next and final steps.

Lastly, if your institution or institutions have a press office, please let them know about your upcoming paper now to help maximize its impact. If they'll be preparing press materials, please inform our press team within the next 48 hours. Your manuscript will remain under strict press embargo until 2 pm Eastern Time on the date of publication. For more information, please contact onepress@plos.org.

You will receive an invoice from PLOS for your publication fee after your manuscript has reached the completed accept phase. If you receive an email requesting payment before acceptance or for any other service, this may be a phishing scheme. Learn how to identify phishing emails and protect your accounts at https://explore.plos.org/phishing.

If we can help with anything else, please email us at customercare@plos.org.

Thank you for submitting your work to PLOS One and supporting open access.

Kind regards,

PLOS One Editorial Office Staff

on behalf of

Dr. Omid Beiki

Academic Editor

PLOS One
